# The Association between Circulating Carotenoids and Risk of Breast Cancer: A Systematic Review and Dose–Response Meta-Analysis of Prospective Studies

**DOI:** 10.1016/j.advnut.2023.10.007

**Published:** 2023-10-30

**Authors:** Maryam Karim Dehnavi, Soraiya Ebrahimpour-Koujan, Keyhan Lotfi, Leila Azadbakht

**Affiliations:** 1 Department of Community Nutrition, School of Nutritional Sciences and Dietetics, Tehran University of Medical Sciences, Tehran, Iran; 2 Students’ Scientific Research Center, Tehran University of Medical Sciences, Tehran, Iran; 3 Department of Clinical Nutrition, School of Nutritional Sciences and Dietetics, Tehran University of Medical Sciences, Tehran, Iran; 4 Autoimmune Bullous Disease Research Center, Razi Hospital, Tehran University of Medical Sciences, Tehran, Iran; 5 Diabetes Research Center, Endocrinology and Metabolism Clinical Sciences Institute, Tehran University of Medical Sciences, Tehran, Iran; 6 Department of Community Nutrition, School of Nutrition and Food Science, Isfahan University of Medical Sciences, Isfahan, Iran

**Keywords:** carotenoids, alpha-carotene, β-carotene, lycopene breast cancer, meta-analysis

## Abstract

Carotenoids appear to have anticancer effects. Prospective evidence for the relation between serum carotenoids and breast cancer is controversial. The present systematic review and meta-analysis aimed to investigate the link between circulating carotenoids and the risk of breast cancer. We performed a systematic search of PubMed, Scopus, and Web of Science up to 30 November, 2022. Prospective studies on adults aged ≥18 y that have reported risk estimates for the association between circulating carotenoids and breast cancer risk were considered. Study quality was assessed using the Newcastle–Ottawa Scale. A random-effects model was used for combining studies’ risk estimates. Dose–response relations were explored through a 1-stage random-effects model. Fifteen publications (17 nested case–control studies and 1 cohort study) with 20,188 participants and 7608 cases were included. We observed an inverse association between the highest level of circulating total carotenoids (relative risk [RR]: 0.76; 95% confidence interval [CI]: 0.62, 0.93; *n* = 8), α-carotene (RR: 0.77; 95% CI: 0.68, 0.87; *n* = 13), β-carotene (RR: 0.80; 95% CI: 0.65, 0.98; *n* = 15), β-cryptoxanthin (RR: 0.85; 95% CI: 0.74, 0.96; *n* = 11), lycopene (RR: 0.86; 95% CI: 0.76, 0.98; *n* = 13), and lutein (RR: 0.70; 95% CI: 0.52, 0.93; *n* = 6) and the risk of breast cancer compared with the lowest level. Additionally, each 10 μg/dL of total carotenoids, α-carotene, β-carotene, and β-cryptoxanthin was associated with 2%, 22%, 4%, and 10% lower risk of breast cancer, respectively. This relationship was stronger at lower levels of total carotenoids and β-cryptoxanthin. The certainty of evidence was rated from very low to low. Most studies were performed among Western nations, which should be acknowledged for extrapolation of findings. Total circulating carotenoids, α-carotene, β-carotene, β-cryptoxanthin, lycopene, and lutein seem to be related to a decreased risk of breast cancer. Our findings could have practical importance for public health.

This study was registered at PROSPERO as CRD42023434983.

**Statement of significance**

According to previous meta-analyses, carotenoids have been found to have anticancer effects. This systematic review and meta-analysis provides a comprehensive review of the association between circulating carotenoids and the risk of breast cancer by considering the most recent prospective studies with large sample sizes and follow-up duration.

## Introduction

Carotenoids are organic pigments made by several plants and bacteria [1]. Of the entire family, α-carotene, β-carotene, lutein/zeaxanthin, lycopene, and β-cryptoxanthin can represent total circulating carotenoids because they make up >95% of the total amount of carotenoids in plasma [1]. Dietary intakes of carotenoids have been suggested to protect human health against various chronic diseases, such as diabetes, cardiovascular disease, stroke, and cancers [2]. Carotenoids can be protective against cancer through their antioxidant functions, as suggested by previous studies [[3], [4], [5]]. Carotenoids can play their anticancer role through DNA protection and repair, singlet oxygen deactivation, suppressing cell proliferation, inducing apoptosis, and inhibiting angiogenesis [[6], [7], [8], [9]].

There are high amounts of carotenoids in fruits and vegetables, especially in yellow/orange fruits and vegetables and green leafy vegetables. Many observational studies have explored the relationship between fruit and vegetable intake and the risk of breast cancer, indicating inconsistent and weak associations [[10], [11], [12], [13]]. A meta-analysis of prospective studies indicated that total fruit and vegetable consumption is associated with lower risk of overall and postmenopausal breast cancer [14]. Studies show a significant correlation between fruit and vegetable intake with plasma carotenoids; hence plasma carotenoids can be a reliable biomarker for fruit and vegetable intake [10,15].

So far, 2 meta-analyses have investigated the relationship between carotenoids and breast cancer [16,17]. Aune et al. [16] compared dietary intake with blood concentrations of carotenoids in relation to breast cancer using prospective observational studies. They found blood concentrations of total carotenoid, lutein, β-carotene, and α-carotene to be strongly associated with a lower risk of breast cancer. Also, dietary β-carotene intake was inversely related to breast cancer, whereas other carotenoids intake did not show a significant association. Another meta-analysis on case–control and prospective cohort studies showed that dietary α-carotene intake is inversely associated with breast cancer risk, and the association between dietary β-carotene and breast cancer risk was marginally significant [17]. However, the authors found no significant association between other dietary carotenoids and breast cancer risk. After the latest meta-analysis, new prospective studies with large sample sizes have been published on circulating carotenoids and showed inconsistent results [9,18]. Therefore, it is relevant to conduct a systematic review and meta-analysis to summarize existing prospective studies exploring the relationship between plasma carotenoids and breast cancer risk and update the previous meta-analysis.

## Methods

### Search strategy

A systematic search was conducted by 2 independent researchers (MKD and SE) using PubMed, Scopus, and Web of Science up to 30 November, 2022. The detailed search strategy is provided in Supplemental Table 1. In brief, the following terms were used in the search strategy to identify observational studies considering carotenoids and breast cancer risk: “Carotenoids,” “α-carotene,” “β-carotene,” “Lycopene,” “Lutein,” “Cryptoxanthin,” “zeaxanthin” AND “breast cancer,” “Breast Neoplasms,” “breast carcinoma,” and “mammary cancer.” To avoid missing any relevant articles, we conducted a manual search of included articles’ reference lists. We also performed a manual web-based search in Google Scholar using combination of “carotenoids” and “breast cancer.” No time or language restriction was applied. Any disagreement was solved by consulting the principal researcher (LA). We followed the PRISMA guidelines. The study was registered at http://www.crd.york.ac.uk/Prospero (registration no. CRD42023434983).

### Study selection

Papers were eligible if they had the following inclusion criteria: *1*) prospective cohort, case–cohort or nested case–control design, *2*) investigated the relationship between total and different types of carotenoids level and breast cancer risk, *3*) reported relative risk (RR) estimates (including RR, odds ratio, hazard ratio, or sufficient information to estimate RR), *4*) carried out on the general population. Letters, comments, reviews, meta-analyses, ecologic studies, Mendelian randomization, and studies that were on populations with a previous history of breast cancer were excluded. For publications that were from the same cohort studies, we included those with the longest follow-up period or those with the largest number of cases.

### Data extraction

Two independent reviewers (MKD and KL) extracted the following data from eligible articles: first author’s last name, year of publication, country, mean age or age range of participants, follow-up period, study design, sample size, incident cases, exposure (type of carotenoid), laboratory assessment of blood carotenoids, comparison, fully adjusted risk estimates with the 95% confidence intervals (CIs), and studies’ covariate adjustments. Any discrepancy was resolved by consensus.

### Risk of bias and certainty of evidence

We used the Newcastle–Ottawa quality assessment scale to estimate risk of bias among eligible papers. This quality assessment tool contains 8 items in 3 domains of selection (4 points), comparability (2 points), and assessment of outcome (3 points). Each paper could receive a score ranging from 0 to 9. In the present study, a score ≥7 was considered to be high quality.

Certainty of evidence was examined through Grading of Recommendations, Assessment, Development and Evaluations (GRADE) [19]. Four levels exist for rating the level of evidence (very low, low, moderate, and high). In this method, possibility of risk of bias, indirectness, imprecision, and inconsistency could downgrade the level of certainty. To upgrade the level of evidence, dose–response gradient, large effect size, and plausible confounding were considered.

### Statistical analysis

The natural logarithm of RRs and 95% CIs were calculated for the highest compared with the lowest categories of total and specific types of carotenoids. To consider between-study heterogeneity, we used a random-effects model to calculate overall effect size. Cochrane’s Q-test and *I*^2^ estimates were applied to estimate between-study heterogeneity and were indicated as significant if P_Q-test_ <0.05 /or *I*^2^ >50%. Subgroup analyses were performed to identify sources of heterogeneity. Subgroup analyses were conducted when ≥10 studies were included based on: age (<55 y/≥55 y), country (United States/non-United States), adjustment for BMI (yes/no), alcohol intake (yes/no), smoking (yes/no), physical activity (yes/no), dietary variables (yes/no), age at menarche (yes/no), hormone therapy (yes/no), oral contraceptive [OC] use (yes/no), age at first birth (yes/no), age at menopause (yes/no), family history of breast cancer (yes/no), and history of benign breast disease (yes/no). If a study reported risk estimates stratified by menopausal status or other variables, we pooled the risk estimates using a fixed-effects model and then included the pooled risk estimate in the main analysis. Visual inspection of funnel plot asymmetry and Egger’s test were used to examine publication bias. We also conducted sensitivity analysis using a random-effects model to assess the influence of each study on the overall risk estimate.

We conducted a linear dose–response analysis per 10 μg/dL of circulating carotenoids using the method developed by Crippa et al. [20]. According to this method, we used the distribution of breast cancer cases and person-years, median of each category, and the RRs with the variance estimates for ≥2 quantitative exposure categories. For studies that did not report cases or person-y for each category, the total number of cases and person-y were divided by the number of categories. We calculated study-specific linear trends from natural logarithms of RRs and 95% CIs across categories of circulating carotenoids using the mean or median dose of circulating carotenoids. If the circulating level of carotenoids was reported as a range, we assigned the midpoint by calculating the mean of the lower and upper bound. If the lowest or the highest category was open-ended, we considered the same length for the open-ended interval as the adjacent interval. For studies that reported circulating levels of carotenoids in μmol/L, we converted the data to μg/dL by dividing the concentration by 0.0186.

We also investigated a potential nonlinear dose–response relationship using restricted cubic splines with 3 knots at percentiles of 10%, 50%, and 90% of the distribution [21]. The correlation within each set of provided risk estimates was accounted for, and the study-specific estimates were combined by using a 1-stage linear mixed effects meta-analysis [20]. This method, which estimates the study-specific slope lines and combines them to get a total average slope in a single stage, is more accurate, flexible, and efficient than the traditional 2-stage approach. All statistical analyses were performed with Stata, version 17 (Stata Corp). *P* values of <0.05 were considered significant.

### Literature research

**FIGURE 1:**
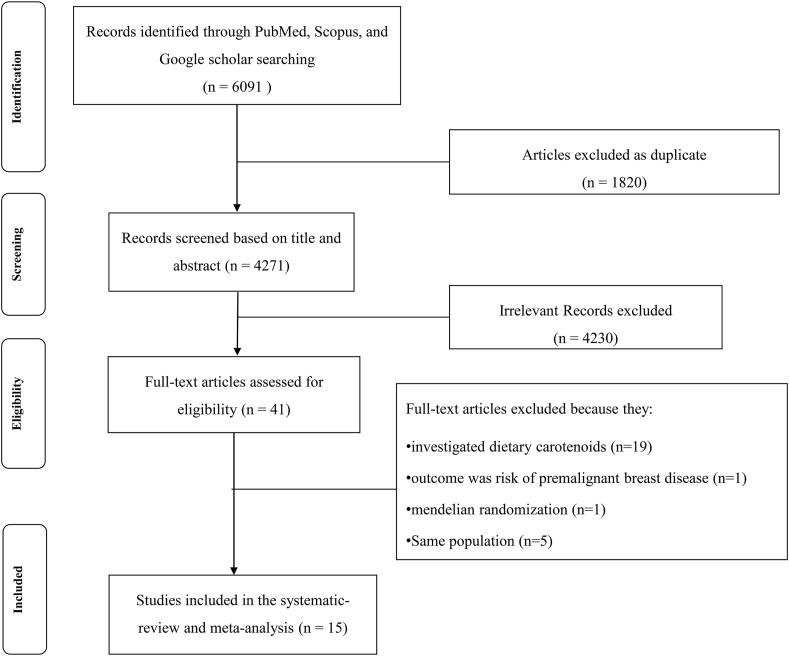
Flow diagram of the study selection process.

In total, 6091 papers were found in the primary search. After exclusion due to being duplicate (*n* = 1820) or not meeting inclusion criteria, 41 papers remained for full-text evaluation. Nineteen studies were excluded because they investigated dietary carotenoids [[22], [23], [24], [25], [26], [27], [28], [29], [30], [31], [32], [33], [34], [35], [36], [37], [38], [39], [40]]. We excluded 1 study because the outcome was the risk of premalignant breast disease rather than breast cancer risk [41]. Also, 1 study was excluded due to being a Mendelian randomization [42]. From 20 studies, 6 studies were based on the Nurses’ Health Study cohort [18,[43], [44], [45], [46], [47]]. We included the most comprehensive study considering duration of follow-up or the number of breast cancer incident cases in the highest compared with the lowest analysis [18]. However, the median values across categories of carotenoids were far divergent from those values reported in other included studies. For example, in Tamimi et al. [46], the median doses of α-carotene ranged from 2.68 to 13.97, and for β-carotene, they ranged from 9.67 to 61.82. In contrast, the Eliassen et al. [18] reported notably higher ranges, with α-carotene spanning from 27.2 to 128.6 and β-carotene ranging from 105 to 487.5. We supposed that these values might not be correct and thus, this study was replaced by another publication of the Nurses’ Health Study cohort [46] for dose–response analysis. Also, from the 2 studies carried out on the European Prospective Investigation into Cancer and Nutrition population, we included the most inclusive study in the present analysis [48]. Finally, we included a total of 15 papers from 17 nested case–control studies and 1 cohort study in the present systematic review and meta-analysis [9,18,35,46,[48], [49], [50], [51], [52], [53], [54], [55], [56], [57], [58]]. One study was excluded from the highest compared with the lowest analysis because RRs and 95% CIs were stated continuously rather than categorically. So, this study was only included in the linear dose–response analysis [49]. One publication reported results separately for 2 different populations; one of them donated blood in 1974 and another one in 1989 [54]. Also, another article had pooled 3 different nested case–control studies and provided 3 effect sizes for pre- and postmenopausal breast cancer, separately [52]. Figure 1 shows the flow diagram of study selection process.

### Study characteristics

The present systematic review and meta-analysis includes 17 nested case–control studies and 1 cohort study (Supplemental Table 2). These studies were published between 1984 and 2016 and had a total number of 20,188 participants. Median follow-up ranged from 8 mo to 21 y during which 7608 breast cancer cases were reported. Four publications did not report the median for follow-up [50,[52], [53], [54]]. Ten studies were from United States [9,18,46,51,[53], [54], [55],^57^,^58^], 5 from Europe (2 of them were reported in 1 article) [48,50,52,59], and 1 from China [56]. All studies assessed circulating carotenoids using high-performance liquid chromatography. The majority of studies carried out on circulating carotenoids and the risk of breast cancer were adjusted for the following variables: BMI (*n* = 9), dietary variables (*n* = 8), age (*n* = 9), alcohol (*n* = 6), age at menarche (*n* = 6), and age at first birth (*n* = 8). Four studies were on postmenopausal women [9,52,57,58], and 11 studies were on pre- and postmenopausal women [18,48,[50], [51], [52], [53], [54], [55], [56],^59^]. According to the quality assessment, except for 2 studies [48,54], other publications had high quality (Supplemental Table 3).

### Total circulating carotenoids

We included 8 effect sizes from 7 publications with 10,863 participants and 5425 breast cancer cases in the analysis of total circulating carotenoids and risk of breast cancer [9,18,48,53,54,56,57]. Findings revealed that the highest levels of total carotenoids compared to the lowest was related to 24% lower risk of breast cancer (RR: 0.76; 95% CI: 0.62, 0.93) (Supplemental Figure 1A). No evidence was observed for between-study heterogeneity (*I*^2^ = 45.6%; *P* = 0.075). According to the sensitivity analysis, no study affected the overall RR. According to Egger’s regression test, publication bias was not significant (*P* = 0.38). Also, no asymmetry was evident according to funnel plot inspection.

**FIGURE 2:**
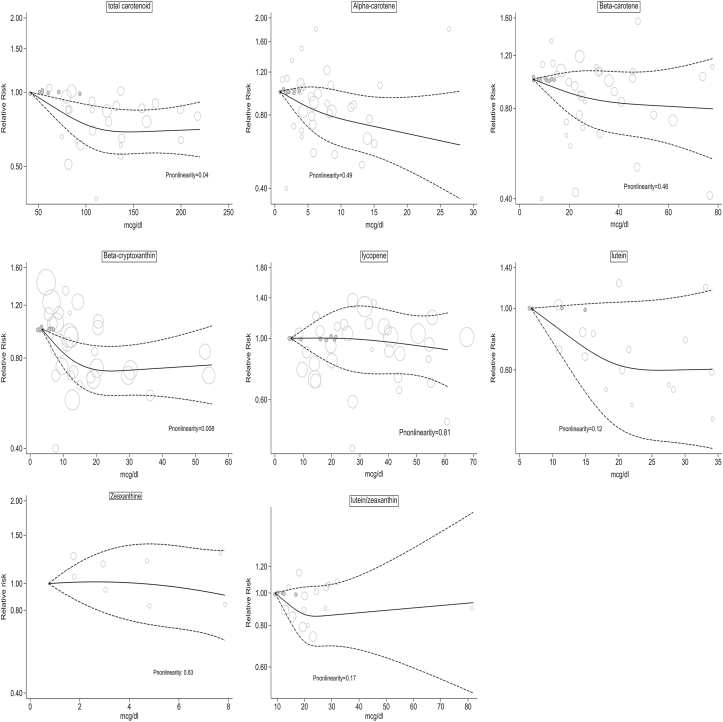
Nonlinear dose–response analysis for the association between circulating carotenoids and risk of breast cancer.

We included 6 studies in dose–response analysis of circulating total carotenoids [9,46,48,54,57]. According to linear dose–response analysis, the risk of breast cancer decreased by 2% for every 10 μg/dL of total carotenoids (RR: 0.98; 95% CI: 0.97, 0.99) (Supplemental Figure 1B). Also, departure from linearity was significant, indicating a nonlinear relationship (*P*-nonlinearity = 0.04). A steady drop in the risk of breast cancer was observed for total carotenoid concentrations <1200 μg/dL followed by a plateau (Figure 2). The level of evidence was graded as low (Supplemental Table 4).

### Circulating α-carotene

We included 13 effect sizes from 11 publications with 18,851 participants and 6630 breast cancer cases in the analysis of circulating α-carotene and risk of breast cancer [9,18,48,[51], [52], [53], [54], [55], [56], [57], [58]]. The highest level of α-carotene, compared with the lowest, was significantly associated with decreased risk of breast cancer (RR: 0.77; 95% CI: 0.68, 0.87), and between-study heterogeneity was not significant (*I*^2^ = 0.0%; *P* = 0.48) (Supplemental Figure 2A). The association between circulating α-carotene and breast cancer risk remained inverse in all subgroups (Table 1). However, findings were not significant in some subgroups, including studies that were in non-US countries, those that did not adjust for BMI, alcohol intake, age at first birth, and hormone therapy, and those that adjusted for physical activity, smoking, and OC use. According to the sensitivity analysis, no study affected the overall RR. According to Egger’s regression test, publication bias did not exist (*P* = 0.58). No asymmetry was evident according to funnel plot as well.

**TABLE 1:** Subgroup analysis for circulating carotenoids and breast cancer risk.

	*n*	RR (95% CI)^1^	*P* within^2^	*I*^2^ (%)	*P* between^3^	*n*	RR (95% CI)^1^	*P* within^2^	*I*^2^ (%)	*P* between^3^
α-carotene						β-carotene				
Overall	13	0.77 (0.68, 0.87)	0.487	0.0		15	0.80 (0.65, 0.98)	0.004	56.5	
Age, y										
<55	6	0.74 (0.58, 0.94)	0.322	14.4	0.755	7	0.77 (0.54, 1.10)	0.003	69.7	0.901
≥55	7	0.78 (0.67, 0.90)	0.474	0.0		8	0.83 (0.64, 1.07)	0.089	43.4	
Country										
US	9	0.78 (0.66, 0.91)	0.311	14.8	0.965	9	0.82 (0.63, 1.06)	0.009	60.9	0.921
Non-US	4	0.76 (0.57, 1.01)	0.549	0.0		6	0.75 (0.50, 1.11)	0.039	57.3	
Adjustment for confounders										
BMI										
Yes	9	0.77 (0.68, 0.89)	0.470	0.0	0.745	9	0.89 (0.72, 1.09)	0.068	45.1	0.004
No	4	0.73 (0.52, 1.01)	0.289	20.2		6	0.60 (0.37, 0.97)	0.005	69.9	
Alcohol										
Yes	6	0.77 (0.67, 0.88)	0.748	0.0	0.984	6	0.90 (0.70, 1.14)	0.029	59.8	0.497
No	7	0.77 (0.56, 1.05)	0.184	31.9		9	0.67 (0.46, 0.96)	0.013	58.5	
Smoking										
Yes	5	0.87 (0.66, 1.15)	0.155	39.9	0.327	5	1.11 (0.77, 1.62)	0.018	66.5	0.004
No	8	0.73 (0.63, 0.85)	0.793	0.0		10	0.68 (0.56, 0.83)	0.215	24.8	
Physical activity										
Yes	3	0.95 (0.72, 1.25)	0.831	0.0	0.087	3	1.17 (0.91, 1.50)	0.459	0.0	0.001
No	10	0.73 (0.63, 0.83)	0.513	0.0		12	0.71 (0.57, 0.87)	0.057	42.8	
Dietary variables										
Yes	8	0.77 (0.61, 0.98)	0.156	34.1	0.876	8	0.93 (0.66, 1.31)	0.004	66.7	0.062
No	5	0.77 (0.66, 0.91)	0.931	0.0		7	0.71 (0.58, 0.87)	0.263	21.8	
Age at menarche										
Yes	6	0.80 (0.69, 0.93)	0.700	0.0	0.487	6	0.89 (0.71, 1.12)	0.030	59.7	0.228
No	7	0.70 (0.54, 0.90)	0.299	17.2		9	0.67 (0.46, 0.98)	0.019	56.4	
Hormone therapy										
Yes	6	0.77 (0.67, 0.88)	0.748	0.0	0.984	6	0.90 (0.70, 1.14)	0.029	59.8	0.497
No	7	0.77 (0.56, 1.05)	0.184	31.9		9	0.67 (0.46, 0.96)	0.013	58.5	
OC use										
Yes	3	0.83 (0.64, 1.09)	0.486	0.0	0.500	3	0.91 (0.61, 1.35)	0.061	64.2	0.550
No	10	0.75 (0.65, 0.87)	0.384	6.3		12	0.76 (0.59, 0.98)	0.006	58.1	
Age at first birth										
Yes	8	0.76 (0.67, 0.87)	0.496	0.0	0.749	8	0.89 (0.70, 1.14)	0.002	68.4	0.094
No	5	0.81 (0.54, 1.23)	0.285	20.3		7	0.62 (0.44, 0.87)	0.302	16.8	
Age at menopause										
Yes	3	0.78 (0.65, 0.92)	0.702	0.0	0.827	3	0.77 (0.65, 0.91)	0.446	0.0	0.663
No	10	0.76 (0.63, 0.93)	0.294	16.2		12	0.78 (0.58, 1.05)	0.001	63.8	
Family history										
Yes	5	0.79 (0.64, 0.97)	0.250	25.8	0.821	6	0.82 (0.58, 1.16)	0.003	72.4	0.620
No	8	0.75 (0.62, 0.91)	0.533	0.0		9	0.77 (0.59, 1.01)	0.086	42.2	
History of benign breast disease										
Yes	5	0.77 (0.60, 1.00)	0.080	52.1	0.639	6	0.84 (0.56, 1.26)	0.001	75.0	0.687
No	8	0.80 (0.65, 0.97)	0.891	0.0		9	0.77 (0.61, 0.97)	0.149	33.6	
**β-cryptoxanthin**						**Lycopene**				
Overall	11	0.85 (0.74, 0.96)	0.807	0.0		13	0.86 (0.76, 0.98)	0.463	0.0	
Age, y										
<55	5	0.77 (0.62, 0.97)	0.782	0.0	0.332	6	0.85 (0.67, 1.07)	0.347	10.8	0.886
≥55	6	0.89 (0.75, 1.04)	0.637	0.0		7	0.87 (0.74, 1.02)	0.405	2.7	
Country										
US	8	0.87 (0.75, 1.00)	0.601	0.0	0.492	9	0.84 (0.74, 0.97)	0.519	0.0	0.484
Non-US	3	0.77 (0.58, 1.03)	0.931	0.0		4	0.95 (0.64, 1.41)	0.247	27.4	
Adjustment for confounders										
BMI										
Yes	8	0.86 (0.75, 0.99)	0.789	0.0	0.441	9	0.91 (0.78, 1.05)	0.381	6.6	0.143
No	3	0.74 (0.52, 1.06)	0.453	0.0		4	0.70 (0.52, 0.95)	0.781	0.0	
Alcohol										
Yes	5	0.88 (0.76, 1.02)	0.615	0.0	0.253	6	0.90 (0.79, 1.04)	0.724	0.0	0.153
No	6	0.73 (0.55, 0.97)	0.832	0.0		7	0.72 (0.54, 0.98)	0.330	13.0	
Smoking										
Yes	4	0.79 (0.63, 1.00)	0.676	0.0	0.524	5	0.89 (0.71, 1.11)	0.752	0.0	0.733
No	7	0.87 (0.74, 1.02)	0.654	0.0		8	0.85 (0.69, 1.06)	0.203	28.3	
Physical activity										
Yes	2	1.02 (0.73, 1.41)	0.366	0.0	0.222	3	1.01 (0.77, 1.32)	0.523	0.0	0.196
No	9	0.82 (0.71, 0.94)	0.875	0.0		10	0.83 (0.72, 0.95)	0.454	0.0	
Dietary variables										
Yes	7	0.76 (0.62, 0.94)	0.852	0.0	0.202	8	0.87 (0.71, 1.07)	0.412	2.4	0.899
No	4	0.91 (0.77, 1.08)	0.610	0.0		5	0.86 (0.71, 1.04)	0.330	13.1	
Age at menarche										
Yes	4	0.87 (0.74, 1.01)	0.498	0.0	0.506	6	0.89 (0.78, 1.03)	0.718	0.0	0.298
No	7	0.79 (0.61, 1.01)	0.773	0.0		7	0.75 (0.55, 1.03)	0.251	23.3	
Hormone therapy										
Yes	5	0.88 (0.76, 1.02)	0.615	0.0	0.253	6	0.90 (0.79, 1.04)	0.724	0.0	0.153
No	6	0.73 (0.55, 0.97)	0.832	0.0		7	0.72 (0.54, 0.98)	0.330	13.0	
OC use										
Yes	3	0.88 (0.68, 1.14)	0.308	15.0	0.677	3	1.03 (0.81, 1.32)	0.675	0.0	0.093
No	8	0.83 (0.71, 0.97)	0.828	0.0		10	0.81 (0.70, 0.94)	0.516	0.0	
Age at first birth										
Yes	6	0.88 (0.76, 1.02)	0.728	0.0	0.281	8	0.89 (0.78, 1.02)	0.677	0.0	0.189
No	5	0.73 (0.54, 0.98)	0.714	0.0		5	0.72 (0.49, 1.05)	0.267	23.1	
Age at menopause										
Yes	2	0.93 (0.73, 1.19)	0.232	30.0	0.249	3	0.91 (0.73, 1.12)	0.267	24.2	0.650
No	9	0.78 (0.65, 0.94)	0.911	0.0		10	0.84 (0.69, 1.01)	0.442	0.0	
Family history										
Yes	4	0.90 (0.76, 1.07)	0.629	0.0	0.297	5	0.89 (0.73, 1.08)	0.330	13.2	0.743
No	7	0.78 (0.64, 0.95)	0.774	0.0		8	0.84 (0.69, 1.02)	0.421	1.1	
History of benign breast disease										
Yes	4	0.85 (0.71, 1.02)	0.679	0.0	0.922	5	0.82 (0.69, 0.97)	0.515	0.0	0.357
No	7	0.84 (0.70, 1.01)	0.599	0.0		8	0.91 (0.75, 1.11)	0.362	8.9	

Abbreviations: BMI, body mass index; CI, confidence interval; OC, oral contraceptive; RR, relative risk.

1 Obtained from random-effects model.

2 *P* for heterogeneity, within subgroup. Obtained by random-effects model.

3 *P* for heterogeneity, between subgroups. Obtained by fixed-effects model.

We included 10 effect sizes from 9 publications in dose–response analysis of circulating α-carotene [9,46,48,49,51,54,55,57,58]. According to linear dose–response analysis, the risk of breast cancer decreased by 22% for every 10 μg/dL of total carotenoids (RR: 0.78; 95% CI: 0.66, 0.93) (Supplemental Figure 2B). We found no evidence for nonlinear association (*P*-nonlinearity = 0.49) (Figure 2). The level of evidence was graded as low (Supplemental Table 4).

### Circulating β-carotene

We included 15 effect sizes from 13 publications with 19,161 participants and 6736 breast cancer cases in the analysis of circulating β-carotene and risk of breast cancer [9,18,48,[50], [51], [52], [53], [54], [55], [56], [57], [58], [59]]. We found a significant inverse association between the highest level of β-carotene and breast cancer risk (RR: 0.80; 95% CI: 0.65, 0.98) (Supplemental Figure 3A). Between-study heterogeneity was significant (*I*^2^ = 56.5%; *P* = 0.004). According to the subgroup analysis, the source of heterogeneity could be adjustment of covariates including BMI, smoking, and physical activity (Table 1). The association between circulating β-carotene and breast cancer risk remained inverse in all strata. However, the results did not remain statistically significant in some subgroups. According to the sensitivity analysis, no study affected the overall effect size. According to Egger’s regression test, publication bias was not evident (*P* = 0.87). Also, no asymmetry was observed in funnel plot.

We included 10 effect sizes from 9 publications in dose–response analysis of circulating β-carotene [9,46,48,49,51,54,55,57,58]. According to linear dose–response analysis, the risk of breast cancer decreased by 4% for every 10 μg/dL of total carotenoids (RR: 0.96; 95% CI: 0.93, 0.99) (Supplemental Figure 3B). We found no evidence for nonlinear association (*P*-nonlinearity = 0.46) (Figure 2). The level of evidence was graded as low (Supplemental Table 4).

### Circulating β-cryptoxanthin

We included 11 effect sizes from 9 publications with 17,347 participants and 5979 breast cancer cases in the analysis of circulating β-cryptoxanthin and risk of breast cancer [9,18,48,51,[53], [54], [55],^57^,^58^]. The summary RR for the highest compared with the lowest level was 0.85 (95% CI: 0.74, 0.96), and between-study heterogeneity was not significant (*I*^2^ = 0.0%; *P* = 0.80) (Supplemental Figure 4A). According to subgroup analyses, the inverse association remained significant among studies that had adjusted BMI and dietary variables and those without adjustment for alcohol intake, physical activity, hormone therapy, OC use, age at first birth, menopause age, and family history (Table 1). Also, the relation was inversely associated among younger populations (mean age <55 y). According to the sensitivity analysis, no study affected the overall risk estimate. According to Egger’s regression test, publication bias was not evident (*P* = 0.43). No asymmetry was detected through funnel plot.

We included 9 effect sizes from 8 publications in dose–response analysis of circulating β-cryptoxanthin [9,46,48,49,51,54,55,58]. According to linear dose–response analysis, the risk of breast cancer decreased by 10% for every 10 μg/dL of total carotenoids (RR: 0.90; 95% CI: 0.82, 0.99) (Supplemental Figure 4B). Also, departure from linearity was significant, indicating a nonlinear relationship (*P*-nonlinearity = 0.008) in which there is a decreasing slope up to the dose of 22 μg/dL followed by a plateau (Figure 2). The level of evidence was graded as low (Supplemental Table 4).

### Circulating lycopene

We included 13 effect sizes from 11 publications with 18,854 participants and 6630 breast cancer cases in the analysis of circulating lycopene and risk of breast cancer [9,18,48,[51], [52], [53], [54], [55], [56], [57], [58]]. The pooled RR was 0.86 (95% CI: 0.76, 0.98) for the highest compared with the lowest category of circulating lycopene (Supplemental Figure 5A). Between-study heterogeneity was not significant (*I*^2^ = 0.0%; *P* = 0.46). According to subgroup analyses, the inverse association remained significant among studies that had adjusted for history of benign breast disease and those without adjustment for BMI, alcohol intake, physical activity, hormone therapy, and OC use. Also, the relation was inversely associated among studies from the United States (Table 1). According to the sensitivity analysis, no study affected the overall risk estimate. According to Egger’s regression test, publication bias was not detected (*P* = 0.43). No asymmetry was observed in funnel plot.

We included 10 effect sizes from 9 publications in dose–response analysis of circulating lycopene [9,46,48,49,51,54,55,57,58]. No significant linear association was found between circulating lycopene and the risk of breast cancer (RR: 0.99; 95% CI: 0.95, 1.02) (Supplemental Figure 5B). Also, the analysis did not show a significant nonlinear association (*P*-nonlinearity = 0.81) (Figure 2). The level of evidence was graded as very low (Supplemental Table 4).

### Circulating lutein

We included 6 effect sizes from 4 publications with 5244 participants and 2533 breast cancer cases in the analysis of total circulating lutein and risk of breast cancer [48,[52], [53], [54]]. The summary RR was 0.70 (95% CI: 0.52, 0.93) for the highest compared with the lowest category of circulating lutein (Supplemental Figure 6A), and between-study heterogeneity was not significant (*I*^2^ = 17.1%; *P* = 0.30). According to the sensitivity analysis, no study affected the overall result. According to Egger’s regression test, publication bias was not detected (*P* = 0.70). No asymmetry was observed in funnel plot as well.

We included 4 effect sizes from 3 publications in dose–response analysis of circulating lutein [48,49,54]. We did not find a significant linear association between circulating lutein and the risk of breast cancer (RR: 0.91; 95% CI: 0.78, 1.05) (Supplemental Figure 6B). Also, the analysis did not show a significant nonlinear association (*P*-nonlinearity = 0.12) (Figure 2). The level of evidence was graded as low (Supplemental Table 4).

### Circulating zeaxanthin

We included 4 effect sizes from 3 publications with 4526 participants and 2174 breast cancer cases in the analysis of total circulating zeaxanthin and risk of breast cancer [48,52,53]. We did not observe a significant relationship between the highest category of circulating zeaxanthin and risk of breast cancer (RR: 0.94; 95% CI: 0.69, 1.28) compared with the lowest (Supplemental Figure 7). Between-study heterogeneity was not significant (*I*^2^ = 0.0%; *P* = 0.56). According to the sensitivity analysis, overall RR did not depend on a single study. According to Egger’s regression test, publication bias was not evident (*P* = 0.99). Funnel plot did not show any evidence of asymmetry as well.

We did not perform a linear dose–response analysis for circulating zeaxanthin and breast cancer risk due to lack of eligible articles. We found no evidence for nonlinear association (*P*-nonlinearity = 0.83) (Figure 2). The level of evidence was graded as very low (Supplemental Table 4).

### Circulating lutein/zeaxanthin

We included 5 effect sizes from 5 publications with 12,063 participants and 3446 breast cancer cases in the analysis of total circulating lutein/zeaxanthin and risk of breast cancer [9,18,51,55,58]. We did not observe a significant relationship comparing the highest compared with the lowest category of circulating lutein/zeaxanthin and risk of breast cancer (RR: 0.90; 95% CI: 0.77, 1.08) (Supplemental Figure 8A). Between-study heterogeneity was not significant (*I*^2^ = 0.0%; *P* = 0.84). According to the sensitivity analysis, no study affected the overall risk estimate. According to Egger’s regression test, publication bias was not detected (*P* = 0.99). No asymmetry was evident according to funnel plot.

We included 5 effect sizes from 5 articles in dose–response analysis [9,46,51,55,58]. There was no evidence of linear association (RR: 0.97; 95% CI: 0.90, 1.05) (Supplemental Figure 8B). We also found no evidence for nonlinear association (*P*-nonlinearity = 0.17) (Figure 2). The level of evidence was graded as very low (Supplemental Table 4).

## Discussion

According to this systematic review and meta-analysis, there is a significant negative relationship between the risk of breast cancer and the highest levels of circulating total carotenoids, α-carotene, β-carotene, β-cryptoxanthin, lutein, and lycopene. According to the subgroup analysis, these associations remained inverse for all but were not significant for several subgroups. According to linear dose–response analysis findings, breast cancer risk decreased by 2%, 22%, 4%, and 10% for every 10 g/dL of total carotenoids for α-carotene, β-carotene, and β-cryptoxanthin, respectively. With a steady drop at lower concentrations, a nonlinear relation was found for total carotenoids and β-cryptoxanthin with breast cancer.

Compared with a previous meta-analysis, we showed that the risk of breast cancer is inversely associated with both total carotenoids and β-carotene [16]. The highest compared with the lowest level of circulating β-carotene, β-cryptoxanthin, lycopene, and lutein were also negatively associated with the risk of breast cancer in our meta-analysis, in contrast to previous meta-analysis with fewer participants, cases, and follow-up duration [16]. In a pooled study of 8 prospective studies investigating the relationship between plasma or serum carotenoids and risk of breast cancer, it was shown that α-carotene, β-carotene, and lycopene had a significant negative relationship with breast cancer but that β-cryptoxanthin did not [60]. Additionally, departure from linearity was significant for total carotenoids and β-cryptoxanthin and the risk of breast cancer, indicating that a gradual risk reduction might occur at lower levels of carotenoids, whereas the previous meta-analysis did not show this result. Similarly, a meta-analysis has demonstrated a nonlinear dose–response relationship with steeper reduction in total cancer risk at lower levels of carotenoids than higher levels [61].

Various fruit and vegetables contain different amounts of carotenoids. Intake of fruit and vegetables has been demonstrated to be connected with greater amounts of circulating carotenoids. [10]. However, studies that examined the circulating level of carotenoids rather than carotenoid intake, including the present study, have shown stronger significant results. Breast cancer risk was found to be inversely correlated with total fruit and vegetable intake and also total fruit intake in a meta-analysis of fruit and vegetable consumption and risk of breast cancer [14]. However, there was no proven link between the risk of breast cancer and tomatoes, cruciferous vegetables, green leafy vegetables, or yellow/orange vegetables. There are 2 meta-analyses and 1 pooled analysis of follow-up studies analyzing the association between dietary intake of carotenoids and the risk of breast cancer [16,17,62]. The pooled analysis found a 5% reduction only for β-cryptoxanthin intake. Between the 2 meta-analyses, only one of them demonstrated a significant negative association for dietary α-carotene and a marginal association for dietary β-carotene. Measurement errors and recall bias while assessing dietary intake and not considering different factors, such as cooking methods that might affect the bioavailability of carotenoids, can explain the weak association between dietary intake of carotenoids and breast cancer risk [63].

Stratified analysis of several factors was performed. Interactions were nonsignificant for most factors. According to our results, heterogeneity was nonsignificant for all carotenoids except β-carotene. There are some ideas for explaining part of this heterogeneity. According to subgroup analyses, *P* for interaction was statistically significant for some subgroups including adjustment for smoking status, BMI, and physical activity. The negative association of β-carotene and breast cancer risk did not remain significant among studies that had adjusted for smoking status. The concentration of carotenoids in blood can become affected and lowered because of oxidative stress caused by smoking [64,65]. Studies have demonstrated that smoking status can significantly moderate the negative relation between circulating β-carotene and risk of breast cancer [51]. Additionally, the negative association of β-carotene and breast cancer risk did not remain significant for the BMI-adjusted subgroup. BMI has an inverse relationship with plasma carotenoids, indicating that women with obesity, the same as smokers, seem to have lower levels of carotenoids in their blood [66]. Women with obesity have a higher oxidative stress so they seem to gain more benefit from carotenoids, although results from a pooled analysis indicated that leaner women have a greater breast cancer risk reduction in relation to carotenoids [60]. The same nonsignificant inverse relation was observed for the physical activity-adjusted subgroup. However, the number of studies that had adjusted for physical activity was too small (*n* = 3). Hence, according to the abovementioned results from subgroup analyses, the negative correlation between β-carotene and breast cancer risk might be due to lack of adjustments for such potential confounders.

There are several underlying mechanisms by which carotenoids can prevent cancer cell formation. One of them is via their antioxidant activity [67]. β-carotene and lycopene are examples of carotenoids that can counteract reactive oxygen species (ROS), avoiding DNA damage and cell mutation that can result in the growth of cancer cells. Additionally, carotenoids can promote the activation of antioxidant enzymes and other cellular defense mechanisms against ROS-induced damage. Some carotenoids have been shown to inhibit cell lines through epigenetic modification and inhibiting lipid peroxidation [68,69]. In addition, gap junction communication is a key factor for controlling cell growth, which can be stimulated and increased by carotenoids [69]. Carotenoids can also interact with Nuclear factor kappa B pathways, impede the production of inflammatory cytokines, and therefore reduce inflammation [70].

The present meta-analysis has several strengths. We minimized selection bias by including only prospective studies with large number of total participants and incident breast cancer cases. To assess a quantitative association, we performed a linear dose–response analysis. By conducting a nonlinear dose–response analysis, we could predict the shape of associations. Moreover, unlike the traditional 2-stage method used in the previous analysis, we employed a 1-stage random-effects model that estimates study-specific slope lines and combines them to derive a single, more accurate, flexible, and efficient total average slope. We also assessed the level of evidence by performing GRADE analysis. Additionally, several subgroup analyses were performed to compare the associations between different populations and also to assess the interaction of carotenoids with different covariates, including history of benign breast disease. Despite the mentioned strengths, some limitations should be considered. First, because carotenoids are fat soluble, their blood level might be affected by the amount and type of fat intake. However, some studies did not adjust for this factor. Due to the limited number of studies considering fat intake in their analyses, we were not able to do a subgroup analysis. Second, we did not report the results stratified by tumor type including estrogen and progesterone receptor because there were few studies reporting results classified by tumor type distinctly. Tumor type might be an important factor because hormonal factors might affect the antioxidant effect of carotenoids [71]. According to the literature, a greater breast cancer risk reduction has been observed for breast cancer cases with estrogen receptor negative (ER–) tumors [60], although experimental studies demonstrated that carotenoids inhibit the growth of both ER– and ER+ cell lines [72]. Third, we could not include all the studies in dose–response analysis due to insufficient data. Fourth, despite finding an inverse relationship between total and most of the subtypes of serum carotenoids and breast cancer risk, the level of evidence was rated as very low to low. This should be taken into account while interpreting the findings. Finally, more than half of the total population was from Western countries so the results should be carefully attributed to other nations.

In summary, according to this systematic review and meta-analysis, there is a significant negative relationship between the risk of breast cancer and the level of circulating total carotenoids, α-carotene, β-carotene, β-cryptoxanthin, lutein, and lycopene. Additionally, every 10 g/dL of total carotenoids, α-carotene, β-carotene, and β-cryptoxanthin was associated with 2%, 22%, 4%, and 10% lower risk of breast cancer, respectively. More prospective studies in various populations with adjustments for potential confounders need to be conducted.

### Author contributions

The authors’ responsibilities were as follows – LA: conception and design; MKD, KL, SE: literature search; MKD, SE: screening; MKD, KL: data extraction; KL, LA: statistical analyses; KL, LA: interpretation of data; MKD, KL SE, LA: drafting of the manuscript; LA: supervised the study; and all authors: read and approved the final manuscript.

### Conflict of interest

The authors report no conflicts of interest.

### Funding

Financial support for conception, design, data analysis, and manuscript drafting comes from Tehran University of Medical Sciences, Tehran, Iran (no. 63955).

### Data availability

The data that support the findings of this study are available from the corresponding author upon reasonable request.

## Appendix A Supplementary data

Multimedia component1 Multimedia component1
