# Supplementary material for: The Association between Circulating Carotenoids and Risk of Breast Cancer: A Systematic Review and Dose–Response Meta-Analysis of Prospective Studies
Source: Adv Nutr. 2023 Oct 30;15(1):100135. doi: 10.1016/j.advnut.2023.10.007 (PMC10694674; doi:10.1016/j.advnut.2023.10.007)
Supplement: Multimedia component1 [file mmc1.docx]

**The association between circulating carotenoids and risk of breast cancer: A systematic review and dose-response meta-analysis of prospective studies**

Maryam Karim Dehnavi et al.

**Supplemental Table 1**: Medical subject headings (MeSH) and non-MeSH keywords used to search relevant publications

| Pubmed  Results: 1792 | (Carotenoids[MeSH] OR Carotenoids[tiab] OR "alpha-carotene" [Supplementary Concept] OR "alpha-carotene"[tiab] OR "beta Carotene"[MeSH] OR "beta Carotene"[tiab] OR Lycopene[MeSH] OR Lycopene[tiab] OR Lutein[MeSH] OR Lutein[tiab] OR Cryptoxanthins[MeSH] OR Cryptoxanthins[tiab] OR Zeaxanthins[MeSH] OR Zeaxanthins[tiab] OR "Vitamin A"[MeSH] OR "Vitamin A"[tiab] OR retinol[tiab]) AND ("breast cancer"[Title/Abstract] OR "Breast Neoplasms"[MeSH Terms] OR "Breast Neoplasms"[tiab] OR "breast carcinoma"[tiab] OR "mammary cancer"[tiab]) |
| --- | --- |
| Scopus  Results: 2397 | (TITLE-ABS-KEY(carotenoids) OR TITLE-ABS-KEY("alpha-carotene") OR TITLE-ABS-KEY("beta Carotene") OR TITLE-ABS-KEY(lycopene) OR TITLE-ABS-KEY(lutein) OR TITLE-ABS-KEY(cryptoxanthins) OR TITLE-ABS-KEY(zeaxanthins) OR TITLE-ABS-KEY("Vitamin A") OR TITLE-ABS-KEY(retinol)) AND (TITLE-ABS-KEY("breast cancer") OR TITLE-ABS-KEY("Breast Neoplasms") OR TITLE-ABS-KEY("breast carcinoma") OR TITLE-ABS-KEY("mammary cancer")) |
| Web of science  Results: 1902 | Carotenoids (Topic) or "alpha-carotene" (Topic) or "beta Carotene" (Topic) or Lycopene (Topic) or Lutein (Topic) or Cryptoxanthins (Topic) or Zeaxanthins (Topic) or "Vitamin A" (Topic) or retinol (Topic) and "breast cancer" (Topic) or "Breast Neoplasms" (Topic) or "breast carcinoma" (Topic) or "mammary cancer" (Topic) |
| Google scholor | carotenoids AND breast cancer |

**Supplemental Table 2:** Main characteristics of prospective cohort studies examining the association between circulating carotenoids and breast cancer risk

| **Author** | **Study name** | **Country** | **Study design** | **Follow-up period, years** | **Study size, menopausal status** | **Age range (mean), years** | **Exposure assessment** | **Exposure (s)** | **Adjustments** |
| --- | --- | --- | --- | --- | --- | --- | --- | --- | --- |
| Bakker et al, 2016 | EPIC | EU | nested case control | 4.1 | 3004 prem and postm: 1502 cases | (49.99) | plasma  HPLC  nmol/L | Total carotenoids  a-Carotene  b-Carotene  b-Cryptoxanthin  Lycopene  Lutein  zeaxanthin | BMI, height, age at menarche, age at first birth, smoking status, alcohol intake, total energy, saturated fatty acids, educational level, season of blood collection, study center, age, menopausal status at recruitment, use of exogenous hormones, phase of menstrual cycle, fasting status at blood collection, time of blood collection |
| Wang et al, 2015 | CPS-II | US | nested case control | 8 | 992 postm; 496 cases | (69.4) | plasma  Reversed-phase HPLC  mcg/L | Total carotenoids  a-Carotene  b-Carotene  b-Cryptoxanthin  Lycopene  Lutein + zeaxanthin | birth date, race/ethnicity, date of blood draw, history of BBD, age at first birth, number of live births, BMI, alcohol consumption, smoking status, menopause hormone therapy use, plasma carotenoids, total fruit and vegetable intake |
| Eliassen et al, 2015 | NHS | US | nested case control | 21 | 4297 postm and prem:2147 cases | 30-55  (56.45) | plasma  Reversed-phase HPLC  mcg/Dl | Total carotenoids  a-Carotene  b-Carotene  b-Cryptoxanthin  Lycopene  Lutein + zeaxanthin | age, menopausal status, postm hormone use, month and time of day, fasting status at blood collection, BMI at age 18 y, weight gain since age 18 y, age at menarche, age at first birth, age at menopause, parity, alcohol intake, history of BBD, family history of breast cancer |
| Pouchieu et al, 2014 | SUVIMAX | France | nested case control | 8 | 200 prem and postm: 100 cases | (49.8) | plasma  HPLC  mcmol/L | a-Carotene  b-Carotene  b-Cryptoxanthin  Lycopene  Lutein | gender, age, number of dietary records, intervention group of the trial, BMI, height, smoking status, physical activity, alcohol intake, educational level, family history of breast cancer, menopausal status, use of hormonal treatment for menopause, number of children, energy, lipid, and fruit and vegetable intakes |
| Kabat et al, 2009 | WHI | US | cohort | 8 | 5450 postm: 190 cases | 50-79  (62.6) | serum  HPLC  mcg/ml | a-Carotene  b-Carotene  b-Cryptoxanthin  Lycopene  Lutein + zeaxanthin | age, education, ethnicity, BMI, OC use, hormone therapy, age at menarche, age at first birth, age at menopause, alcohol, family history of breast cancer, history of breast biopsy, physical activity, energy intake, randomization status in hormone therapy, calcium plus vitamin D, dietary modification trials |
| Epplein et al, 2009 | MEC |  | nested case control | 2001-2006  NR | 821 postm: 286 cases | 45-75  (66) | plasma  HPLC  ng/ml | Total carotenoids  a-Carotene  b-Carotene  Lycopene | birth year, race/ethnicity, location, date and time of blood draw, fasting hours, hormone therapy use, age at blood draw, fasting hours prior to blood draw, body mass index, alcohol use, age at menarche, age at menopause, age at first birth, number of full-term pregnancies |
| Dorjgochoo et al, 2009 | SWHS | China | nested case control | 7.5 | 1091 prem and postm: 365 cases | 40-70  (52.56) | plasma  Reversed-phase HPLC | Total carotenoids  a-Carotene (trans)  b-Carotene  Lycopene | age at baseline, date and time of blood collection, interval since last meal, menopausal status, antibiotic use in the past week, age at entry, education, occupation, age at menarche, age at 1st live birth, waist to hips ratio, exercised regularly in past 5 years, ever smoke, menopausal status, history of breast fibroadenoma, 1st degree family history cancer, total intakes of energy, vegetables, fruit, red meat and fish, regular tea consumption, batch for assays, other plasma lipophilic antioxidants |
| Sesso et al, 2005 | WHS | US | nested case control | 7 | 1016 prem and postm: 508 cases | ≥45  (53.9) | plasma  HPLC | a-Carotene  b-Carotene  b-Cryptoxanthin  Lycopene  Lutein + zeaxanthin | age, smoking status, randomized aspirin treatment, randomized vitamin E treatment, randomized h-carotene treatment, plasma cholesterol level, BMI, family history of breast cancer, physical activity, age at menarche, ever use of OCs, age at first birth, number of pregnancies, postm status, and postm hormone use, alcohol intake, fiber intake, folate intake, saturated fat intake, fruit and vegetable intake |
| Tamimi et al, 2005 | NHS | US | nested case control | 8.5 | 212 prem and 663 postm controls,  206 prem and 666 postm cases | 43-70  (57) | Plasma, HPLC | Total carotenoids  a-Carotene  b-Carotene  b-Cryptoxanthin  Lutein + zeaxanthin | age, menopausal status, postm hormone use, time of day, month, and fasting status at blood draw, history of BBD, family history of breast cancer, age at first birth, parity, age at menopause, age at menarche, alcohol consumption, duration of postm hormone use |
| Sato et al, 2002 | Washington  County  Study | US | nested case control | 1975-1994  1989-1994  NR | 488 prem and posm: 244 cases  230 prem and postm: 115 cases | (51.2)  (60.33) | Serum/plasma  reversed-phase HPLC | Total carotenoids  a-Carotene  b-Carotene  b-Cryptoxanthin  Lycopene  Lutein | age, race, menopausal status, month and year of blood donation, prem women on date of last menstrual cycle. |
| Toniolo et al, 2001 | New York University Women’s Health Study | US | nested case control | 1985–1994  NR | 540 prem and postm; 270 cases | 35-65  (51.95) | serum  HPLC | Total carotenoids  a-Carotene  b-Carotene  b-Cryptoxanthin  Lycopene  Lutein  zeaxanthin | age at recruitment, menopausal status at baseline, date of baseline blood sampling, number of blood samplings prior to a case’s date of diagnosis, phase and day of the menstrual cycle at baseline, age at first full-term pregnancy, family history of breast cancer, history of BBD, total cholesterol |
| Hulten et al, 2001 | MSP  VIP+MONICA | Sweden | nested case±referent | 1995-1997; NR  1985-1997; NR  recruited in 1986, 1990, 1994; NR | 491 postm: 201 cases  491 prem and postm: 201 cases | (59)  (52) | plasma  HPLC | a-Carotene  b-Carotene  b-Cryptoxanthin  Lycopene  Lutein  zeaxanthin | age, date of blood sample, sampling centre, BMI, plasma total cholesterol and triglycerides |
| Dorgan et al, 1998 | The Columbia Missouri Breast Cancer Serum Bank | US | nested case control | 9.5 y follow-up  median=2.7 y | 308 prem and postm: 105 cases | 41-72  (58.49) | serum  reversed-phase HPLC | a-Carotene  b-Carotene  b-Cryptoxanthin  Lycopene  Lutein + zeaxanthin | age, history of BBD diagnosis during prior 2 years, month and year of blood collection, total serum cholesterol concentration, packs of cigarettes smoked/day, BMI |
| Wald et al, 1984 | Guernsey Study | UK | nested case control | 1968/1975–1982; NR | 117 prem and postm: 39 cases | 28-75 | Serum  HPLC | b-Carotene | age, menopausal status, parity, family history of breast cancer, history of BBD, age, duration of storage of serum samples |
| Knekt et al, 1990 | Finnish Mobile Clinic Health Examination Survey | Finland | nested case control | 8 | 190 prem and postm: 67 cases | 15-99 | Serum  HPLC | b-Carotene | age, place of residence, municipality, season of blood withdrawal, duration of storage of the serum samples |

EPIC, The European Prospective Investigation into Cancer and Nutrition; ; postm, postmenopausal; prem, premenopausal; HPLC, High-performance liquid chromatography; OC, use of oral contraceptives; CPS, Cancer Prevention Study; BBD, benign breast disease; NHS, The Nurses' Health Study; SUVIMAX, Supplementation en Vitamines et Mineraux Antioxydants; WHI, Women's Health Initiative; MEC, Multiethnic Cohort Study; NR, not reported; SWHS, Shanghai Women and Men's Health Study; WHS, Women's Health Study; VIP, Va¨sterbotten Intervention Project; MSP, The Mammary Screening; MONICA, Monitoring of Trends in Cardiovascular Disease Project

**Supplemental Table 3:** Quality assessment of prospective cohort studies included in the meta-analysis on circulating carotenoid and breast cancer risk1

|  | Representativeness of the exposed cohort | Selection of the non-exposed cohort | Ascertainment of exposure | Demonstration that outcome of interest was not present at start of study | Study controls for fruit/vegetable intake | Study controls for any additional factor (other variables) | Assessment of outcome | Was follow-up long enough for outcomes to occur (>10 years) | Adequacy of follow up of cohorts (loss-to-follow up <20%) | Total score |
| --- | --- | --- | --- | --- | --- | --- | --- | --- | --- | --- |
| Bakker 2016 | ***** | ***** | ***** | ***** |  | ***** | ***** |  |  | 6 |
| Wang 2015 | ***** | ***** | ***** | ***** | ***** | ***** | ***** |  | ***** | 8 |
| Eliassen 2015 |  | ***** | ***** | ***** |  | ***** | ***** | ***** | ***** | 7 |
| Pouchieu 2014 | ***** | ***** | ***** | ***** | ***** | ***** | ***** |  | ***** | 8 |
| Kabat 2009 | ***** | ***** | ***** | ***** |  | ***** | ***** |  | ***** | 7 |
| Epplein 2009 | ***** | ***** | ***** | ***** |  | ***** | ***** |  | ***** | 7 |
| Dorjgochoo 2009 | ***** | ***** | ***** | ***** | ***** | ***** | ***** |  | ***** | 8 |
| Sesso 2005 |  | ***** | ***** | ***** | ***** | ***** | ***** |  | ***** | 7 |
| Sato 2002 | ***** | ***** | ***** | ***** |  | ***** | ***** |  |  | 6 |
| Toniolo 2001 | ***** | ***** | ***** | ***** |  | ***** | ***** |  | ***** | 7 |
| Hulten 2001 | ***** | ***** | ***** | ***** |  | ***** | ***** |  | ***** | 7 |
| Dorgan 1998 | ***** | ***** | ***** | ***** |  | ***** | ***** |  | ***** | 7 |
| Knekt 1990 | ***** | ***** | ***** | ***** |  | ***** | ***** |  | ***** | 7 |
| Wald 1984 | ***** | ***** | ***** | ***** |  | ***** | ***** | ***** | ***** | 8 |

**^1^**According to the Newcastle-Ottawa Scale (NOS) criteria

**Supplemental Table 4**: GRADE evidence table for the association of circulating carotenoids and risk of breast cancer

| **Certainty assessment** | | | | | | | | | |  | | | **Effect** | | | **Certainty** | **Importance** |
| --- | --- | --- | --- | --- | --- | --- | --- | --- | --- | --- | --- | --- | --- | --- | --- | --- | --- |
| **№ of studies** | **Study design** | **Risk of bias** | **Inconsistency** | | **Indirectness** | **Imprecision** | **Other considerations** | | | **Participants** | | **Cases (Event rate%)** | **Relative (95% CI)** | **Absolute (95% CI)** | |  |  |
| **Total carotenoids** | | | | | | | | | | | | | | | | | |
| **8** | **observational studies** | **not serious** | **not serious** | | **serious^a^** | **not serious** | **dose response gradient** | | | **10863** | | **5425 (49.9%)** | **RR 0.76 (0.62 to 0.93)** | **120 fewer per 1,000 (from 190 fewer to 35 fewer)** | | **⨁⨁◯◯ Low** | **CRITICAL** |
| **Alpha-carotene** | | | | | | | | | | | | | | | | | |
| **13** | **observational studies** | **not serious** | **not serious** | | **serious^a^** | **not serious** | | **dose response gradient** | **18854** | | | **6630 (35.2%)** | **RR 0.77 (0.68 to 0.87)** | **81 fewer per 1,000 (from 113 fewer to 46 fewer)** | | **⨁⨁◯◯ Low** | **CRITICAL** |
| **Beta-carotene** | | | | | | | | | | | | | | | | | |
| **15** | **observational studies** | **not serious** | **not serious^b^** | | **Serious^a^** | **not serious** | | **dose response gradient** | **17320** | | | **6736 (38.9%)** | **RR 0.80 (0.65 to 0.98)** | **78 fewer per 1,000 (from 136 fewer to 8 fewer)** | | **⨁⨁◯◯ Low** | **CRITICAL** |
| **Beta-cryptoxanthin** | | | | | | | | | | | | | | | | | |
| **11** | **observational studies** | **not serious** | | **not serious** | **serious^a^** | **not serious** | | **dose response gradient** | | | **17347** | **5979 (34.5%)** | **RR 0.85 (0.74 to 0.90)** | | **52 fewer per 1,000 (from 90 fewer to 34 fewer)** | **⨁⨁◯◯ Low** | **CRITICAL** |
| **Lycopene** | |  | |  |  |  | |  | | |  |  |  | |  |  |  |
| **13** | **observational studies** | **not serious** | | **not serious** | **serious^a^** | **not serious** | | **none** | | | **17013** | **6630 (39.0%)** | **RR 0.86 (0.76 to 0.98)** | | **55 fewer per 1,000 (from 94 fewer to 8 fewer)** | **⨁◯◯◯ Very low** | **CRITICAL** |
| **Lutein** | |  | |  |  |  | |  | | |  |  |  | |  |  |  |
| **6** | **observational studies** | **not serious** | | **not serious** | **not serious** | **not serious** | | **none** | | | **5244** | **2533 (48.3%)** | **RR 0.70 (0.52 to 0.93)** | | **145 fewer per 1,000 (from 232 fewer to 34 fewer)** | **⨁⨁◯◯ Low** | **CRITICAL** |
| **zeaxanthin** | |  | |  |  |  | |  | | |  |  |  | |  |  |  |
| **4** | **observational studies** | **not serious** | | **not serious** | **not serious** | **serious^c^** | | **none** | | | **4526** | **2174 (48.0%)** | **RR 0.94 (0.69 to 1.28)** | | **29 fewer per 1,000 (from 149 fewer to 134 more)** | **⨁◯◯◯ Very low** | **CRITICAL** |
| **Lutein/zeaxanthin** | | | |  |  |  | |  | | |  |  |  | |  |  |  |
| **5** | **observational studies** | **not serious** | | **not serious** | **serious^a^** | **serious^c^** | | **none** | | | **12063** | **3446 (28.6%)** | **RR 0.90 (0.77 to 1.06)** | | **29 fewer per 1,000 (from 66 fewer to 17 more)** | **⨁◯◯◯ Very low** | **CRITICAL** |

**CI:** confidence interval; **RR:** risk ratio

**Explanations**

a. serious indirectness since more than 50% of population is from US. Downgraded

b. although I^2^=56.5%, we did not downgrade for inconsistency since in the subgroup of unadjusted smoking status studies the RR=0.68 (0.56, 0.83, n=10, I2=24.8%). Not downgraded

c. serious imprecision since 95% CI include the null value (RR=1.00). Downgraded.

**
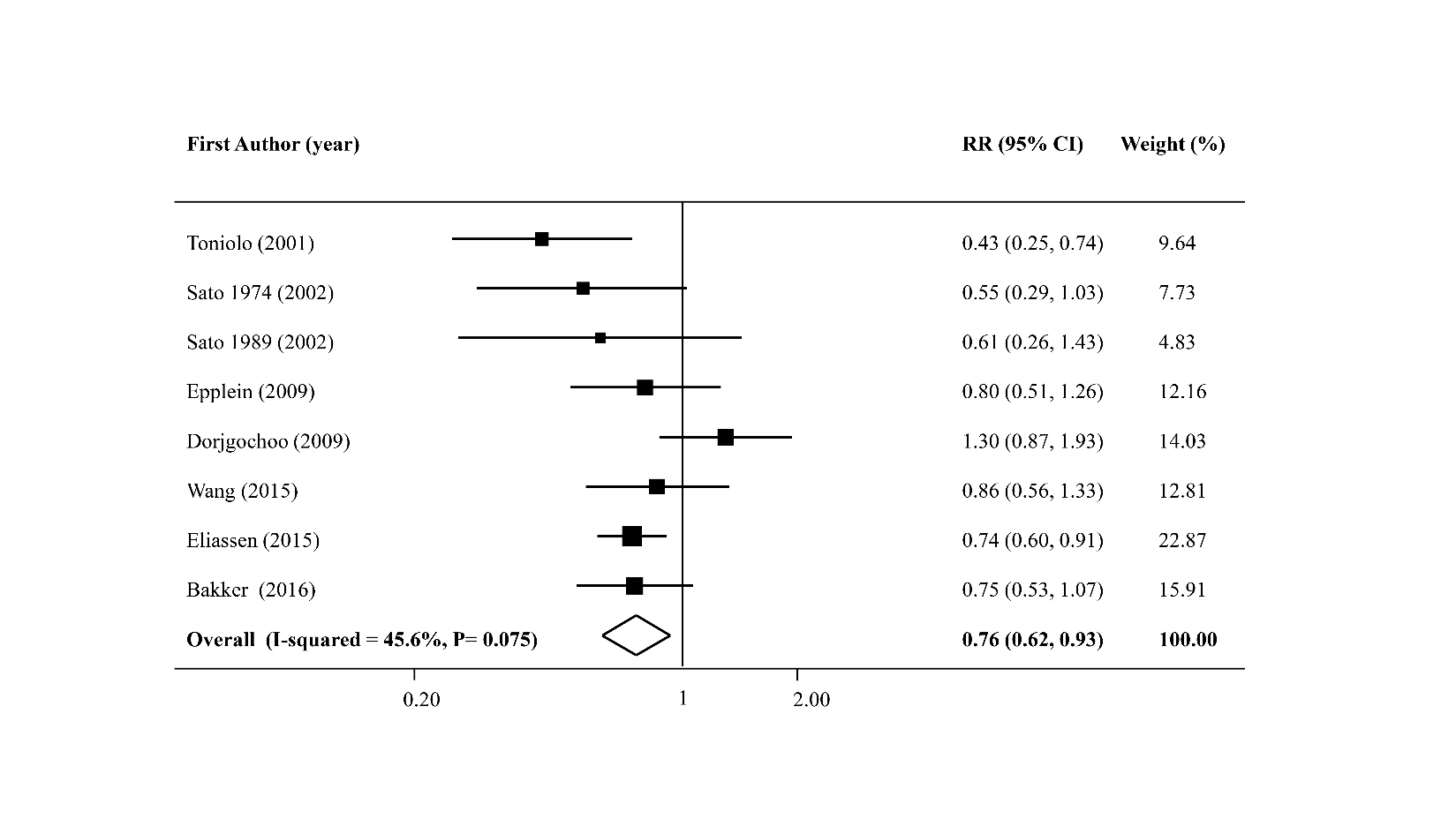
**

**Supplemental Figure 1A**: Forest plot for the association between total circulating carotenoids and risk of breast cancer, expressed as comparison between highest and lowest categories of total circulating carotenoids. Diamonds represent pooled estimates from random effects analysis


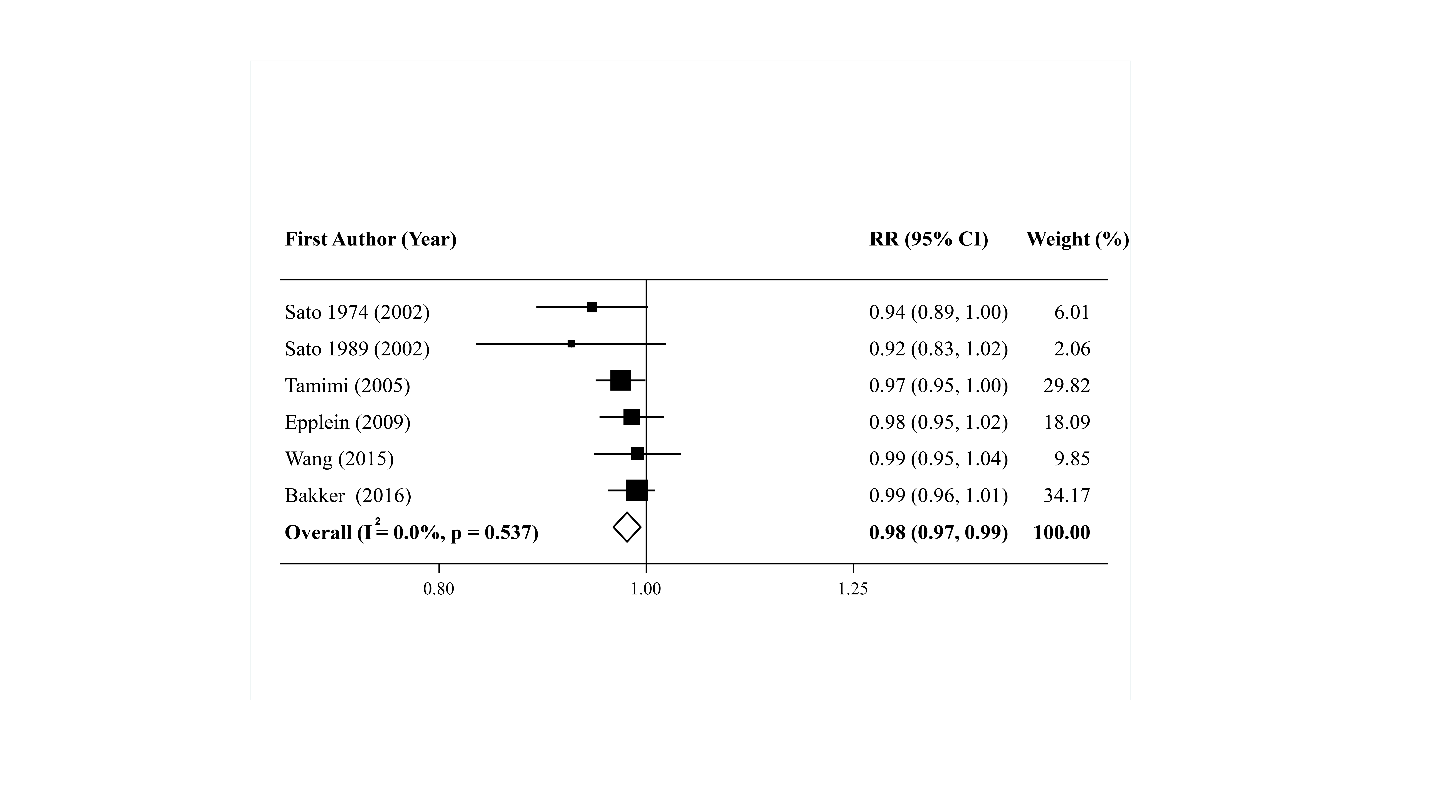


**Supplemental Figure 1B**. Linear dose-response analysis for the association between each additional 10 µg/dl of total circulating carotenoids and risk of breast cancer


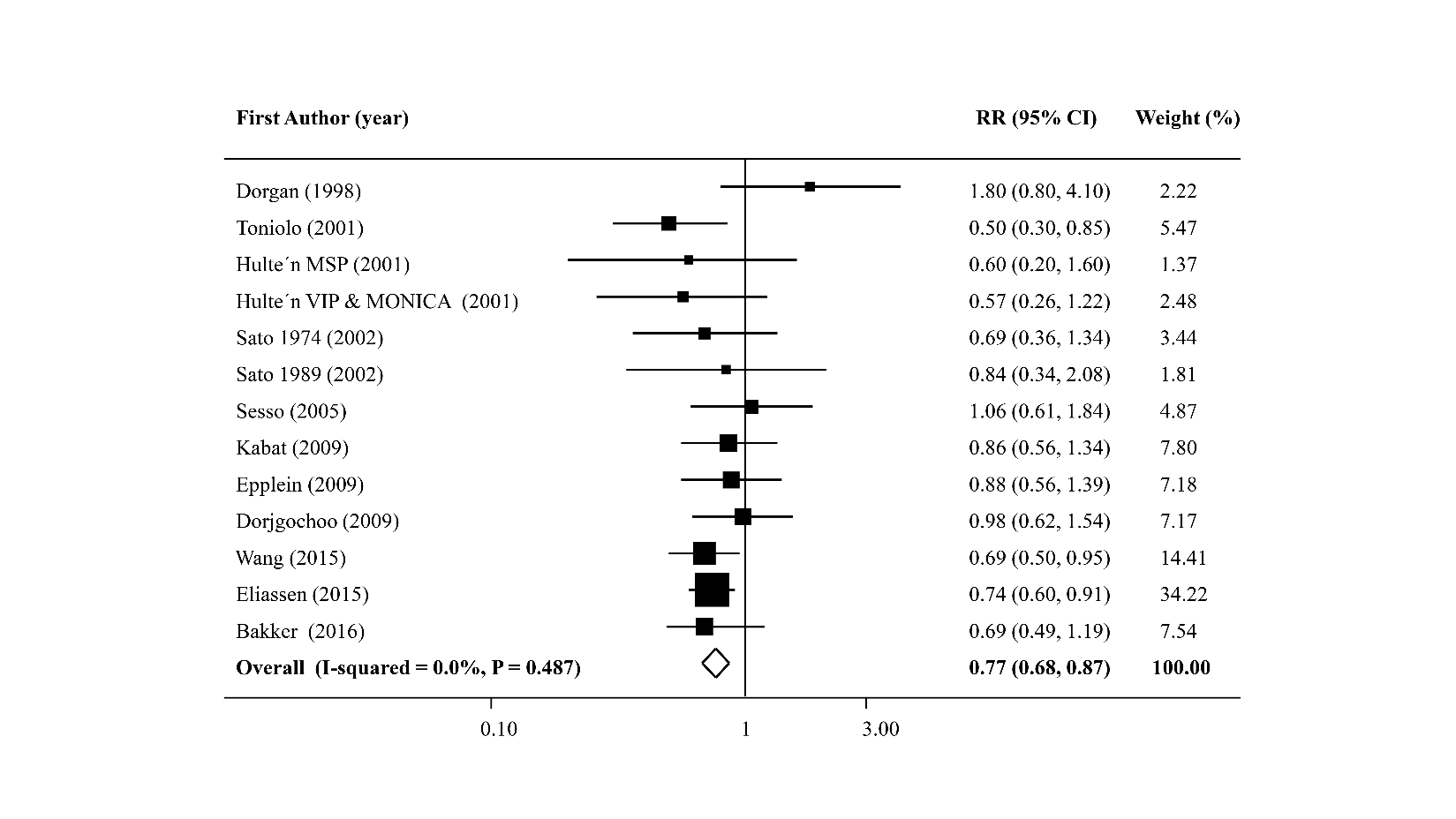
**Supplemental Figure 2A**. Forest plot for the association between circulating α-carotene and risk of breast cancer, expressed as comparison between highest and lowest categories of circulaing α-carotene. Diamonds represent pooled estimates from random effects analysis.

**
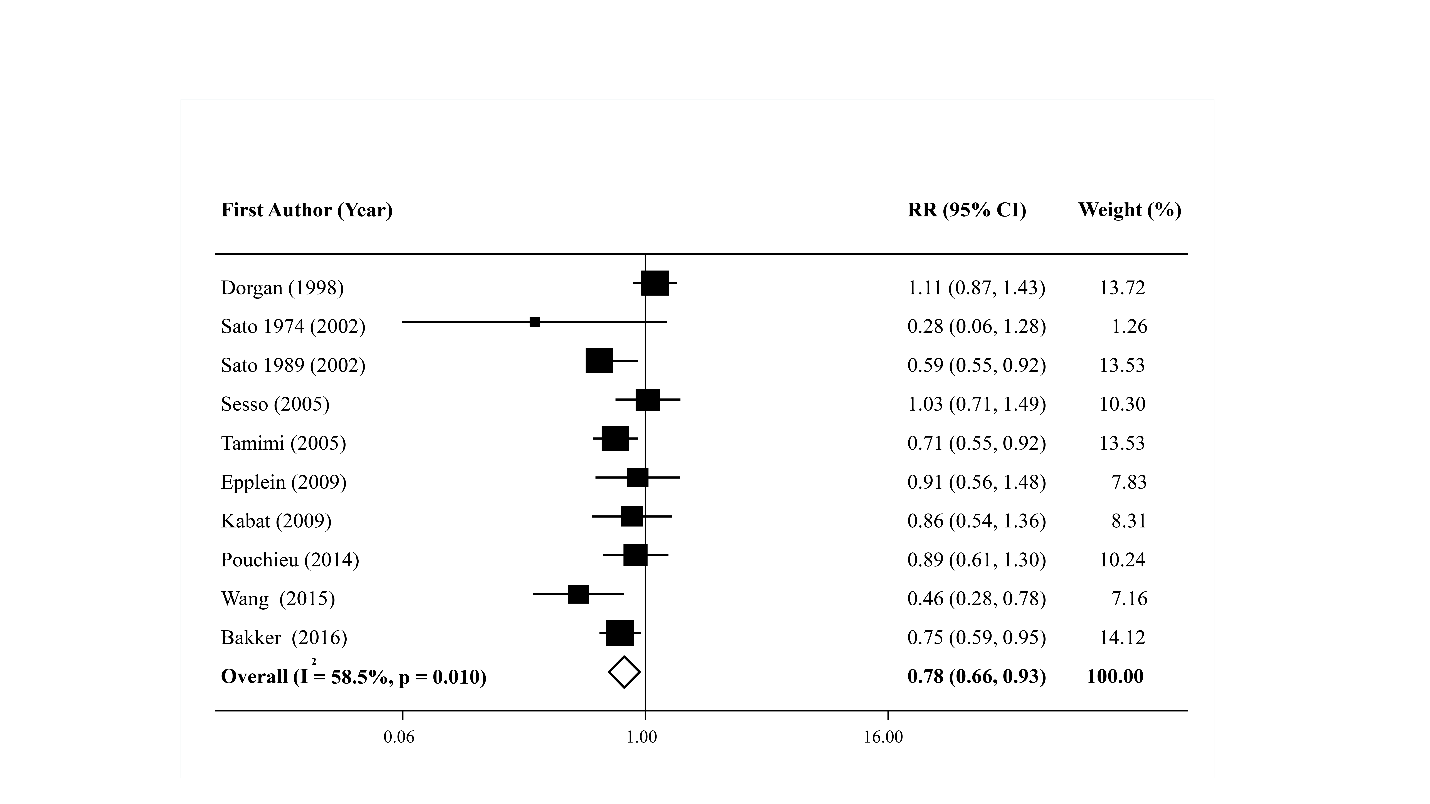
**

**Supplemental** **Figure 2B**. Linear dose-response analysis for the association between each additional 10 µg/dl of circulating α-carotene and risk of breast cancer**.**


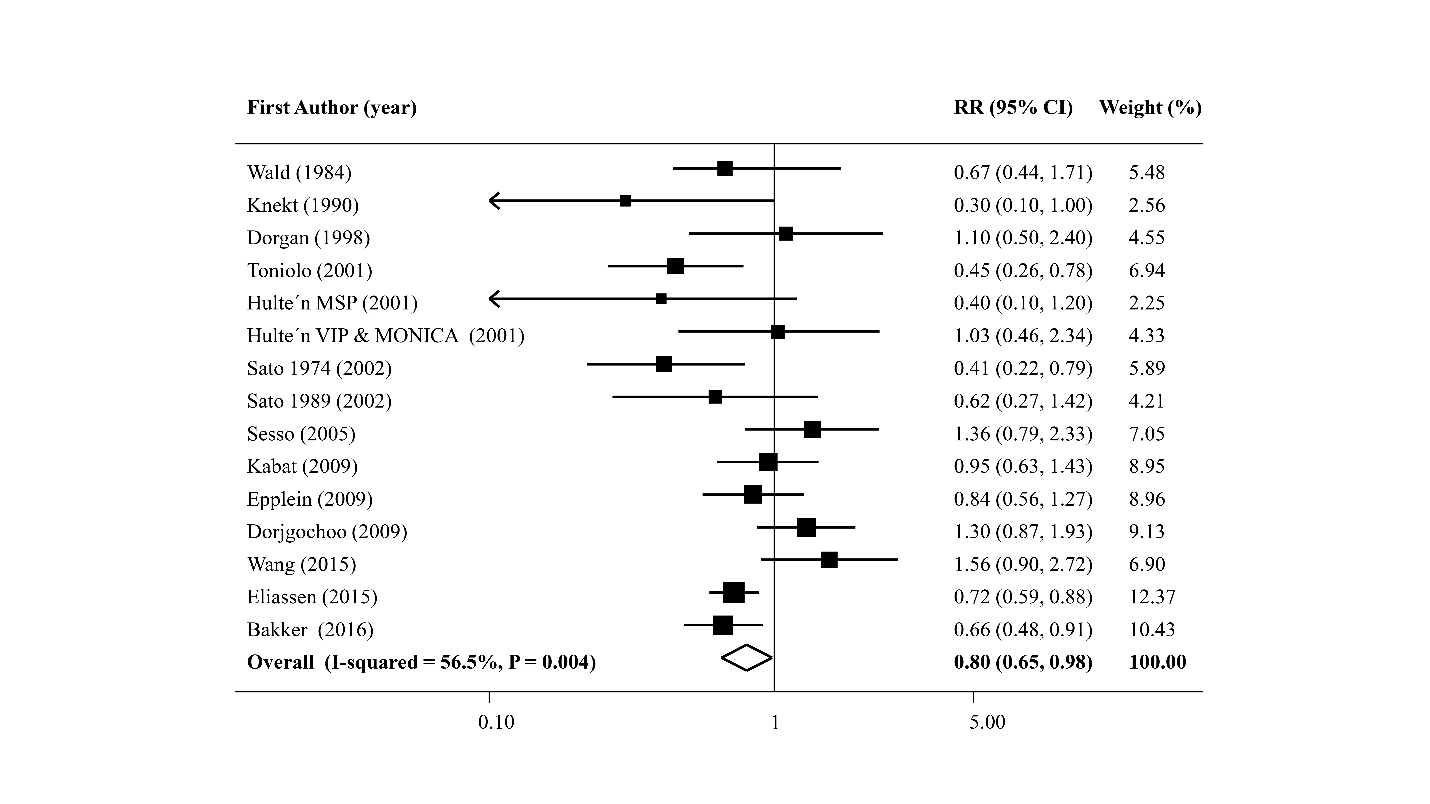
**Supplemental Figure 3A**. Forest plot for the association between circulating β-carotene and risk of breast cancer, expressed as comparison between highest and lowest categories of circulaing β-carotene. Diamonds represent pooled estimates from random effects analysis.


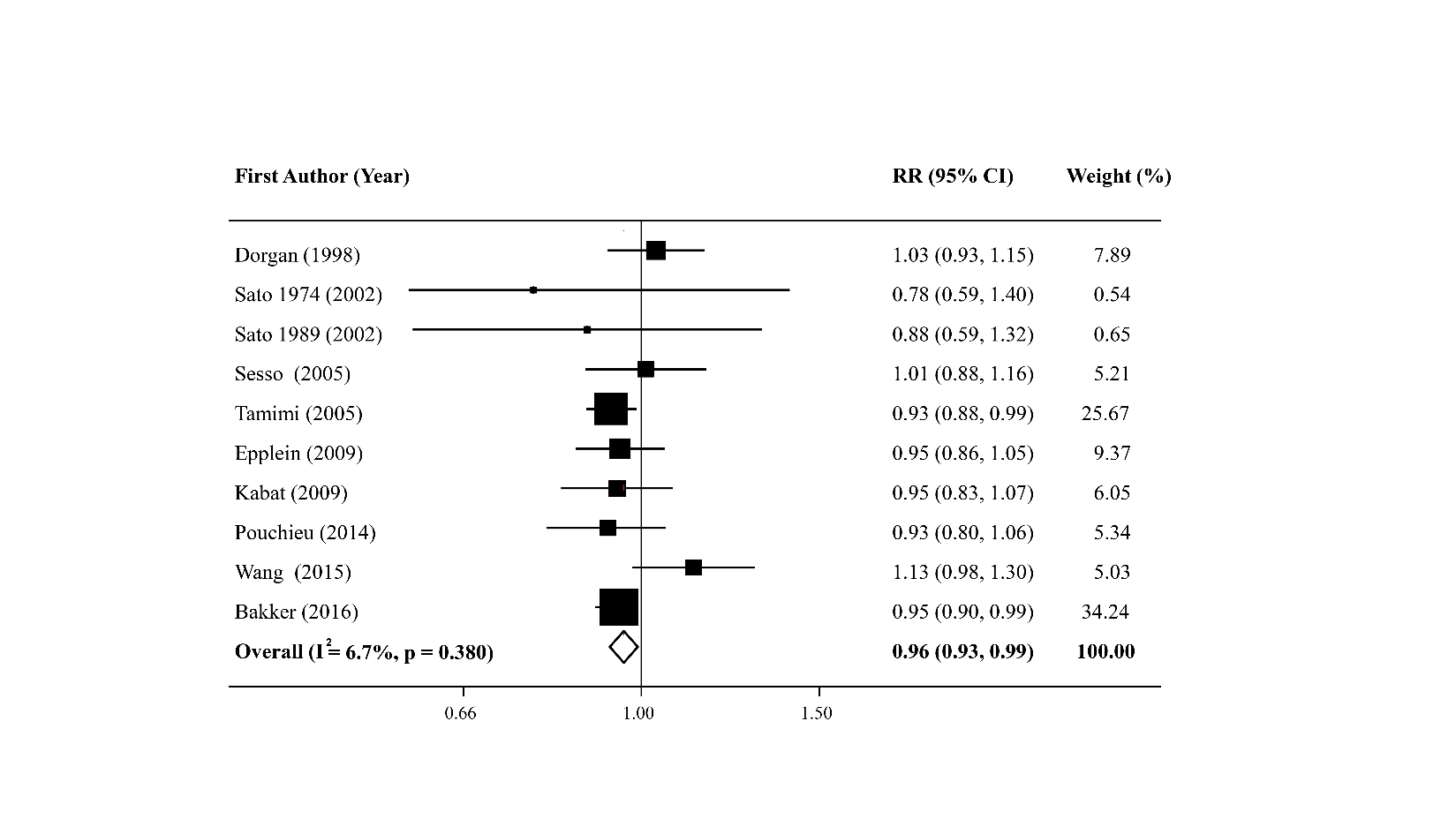


**Supplemental Figure 3B**. Linear dose-response analysis for the association between each additional 10 µg/dl of circulating β-carotene and risk of breast cancer.


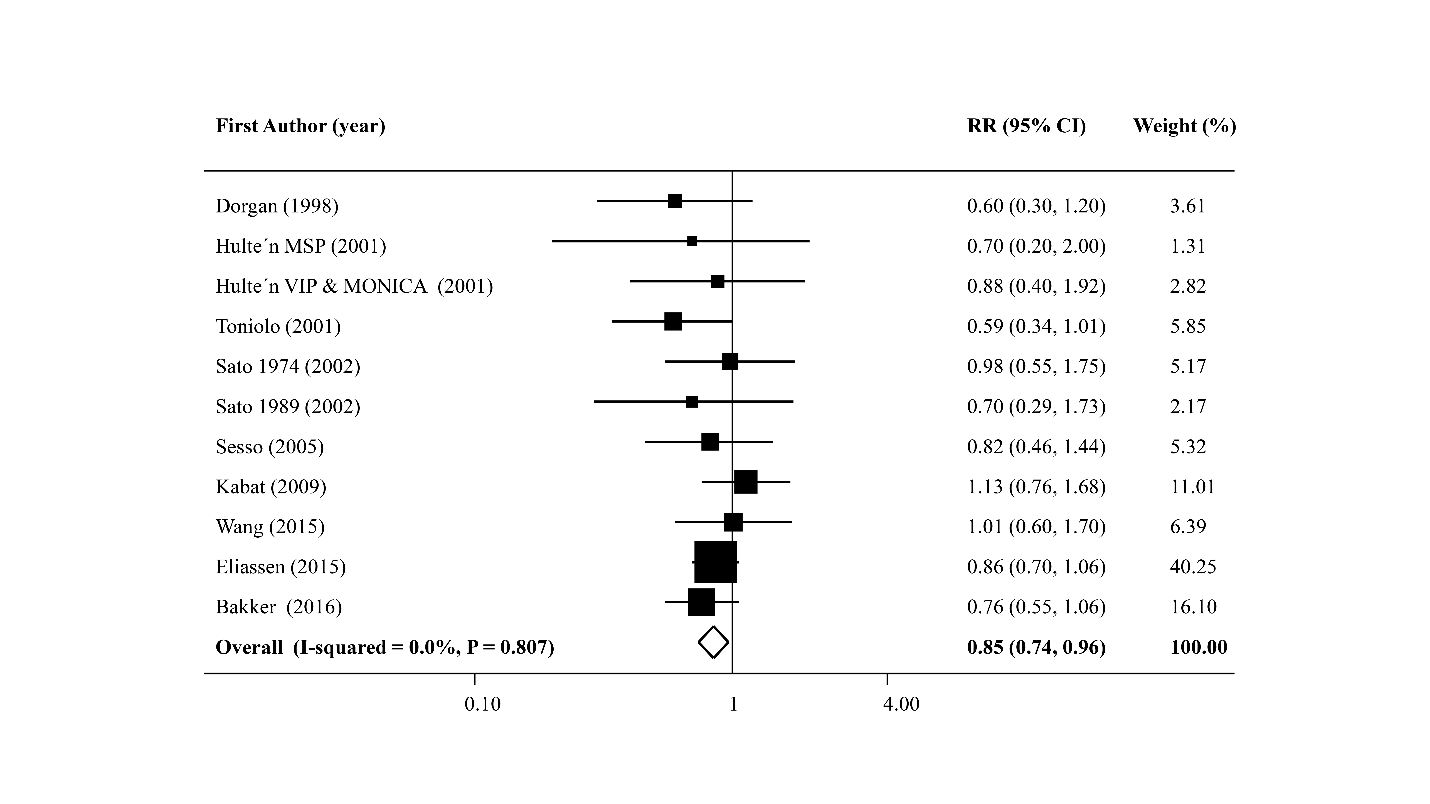
**Supplemental Figure 4A**. Forest plot for the association between circulating β-cryptoxanthin and risk of breast cancer, expressed as comparison between highest and lowest categories of circulaing β-cryptoxanthin. Diamonds represent pooled estimates from random effects analysis.


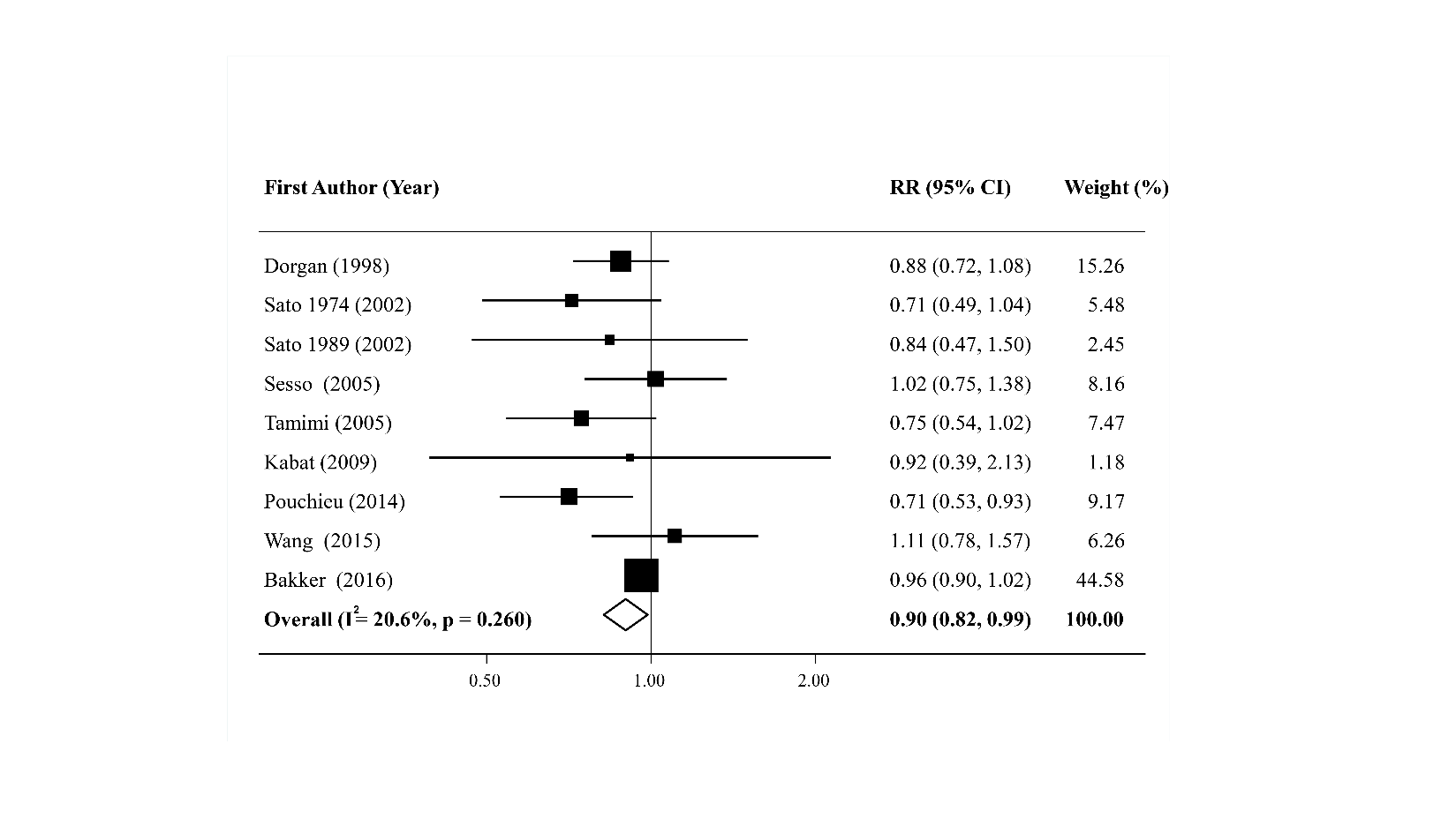


**Supplemental Figure 4B**. Linear dose-response analysis for the association between each additional 10 µg/dl of circulating β-carotene and risk of breast cancer.


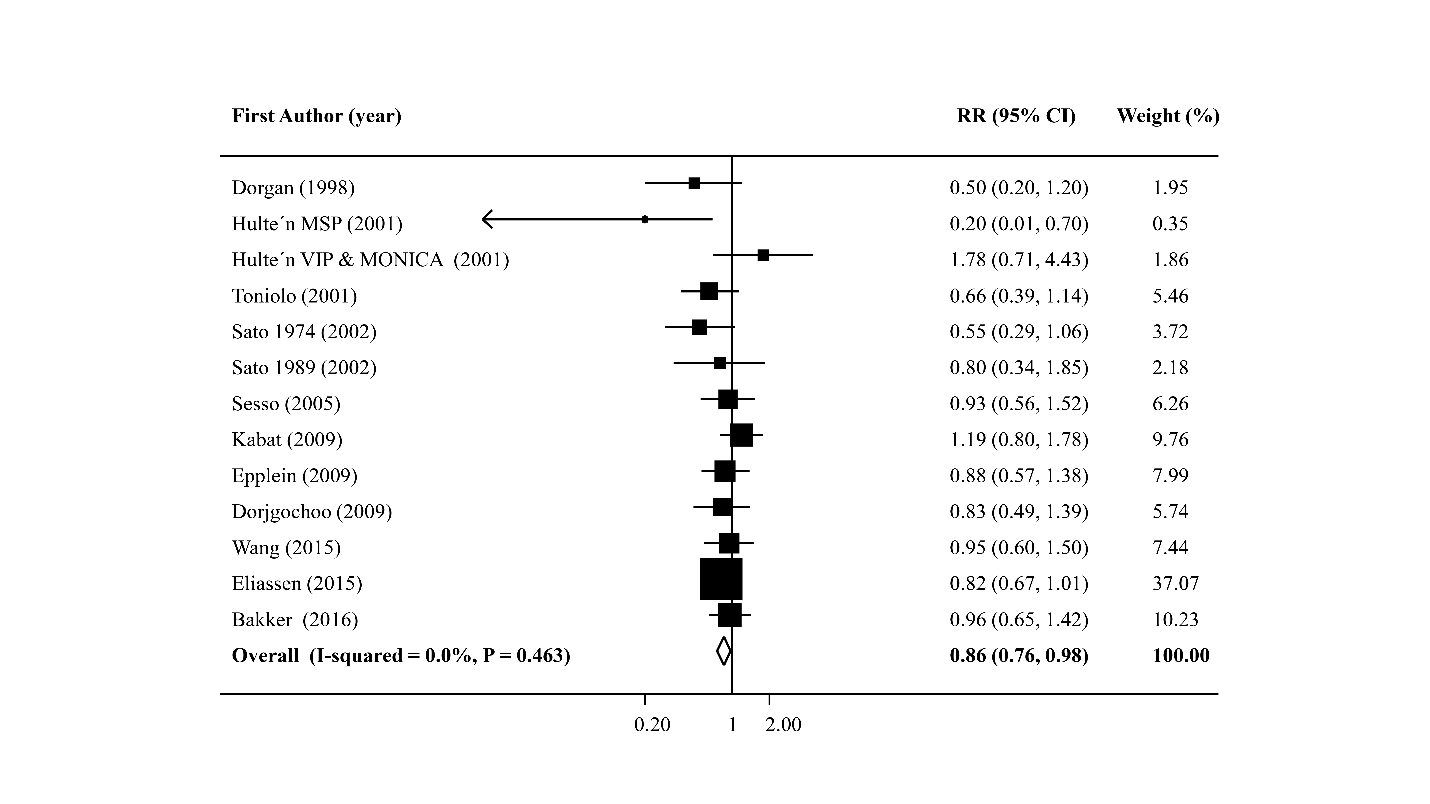
**Supplemental Figure 5A**. Forest plot for the association between circulating lycopene and risk of breast cancer, expressed as comparison between highest and lowest categories of circulaing lycopene. Diamonds represent pooled estimates from random effects analysis.


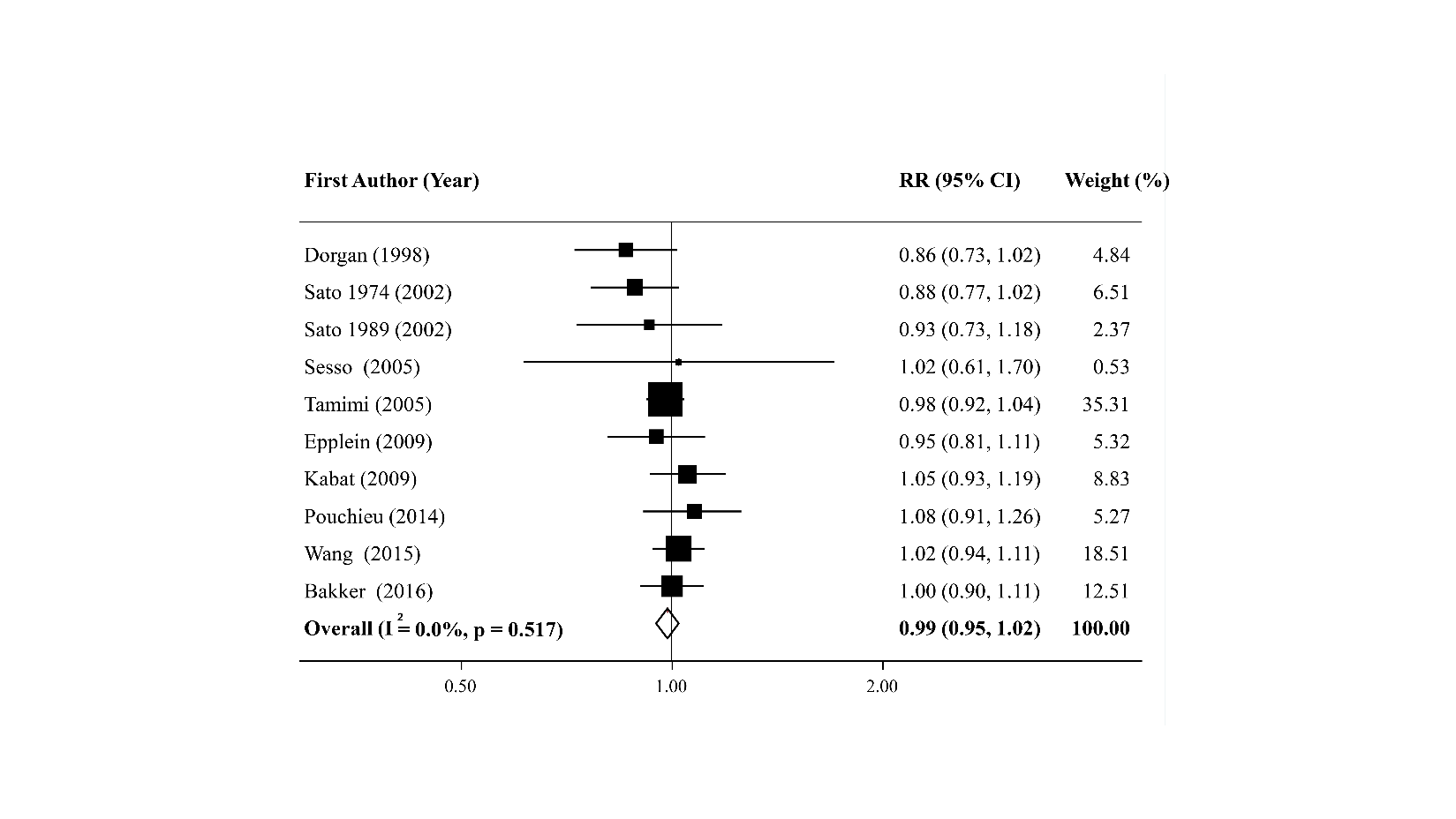


**Supplemental Figure 5B**. Linear dose-response analysis for the association between each additional 10 µg/dl of circulating lycopene and risk of breast cancer.


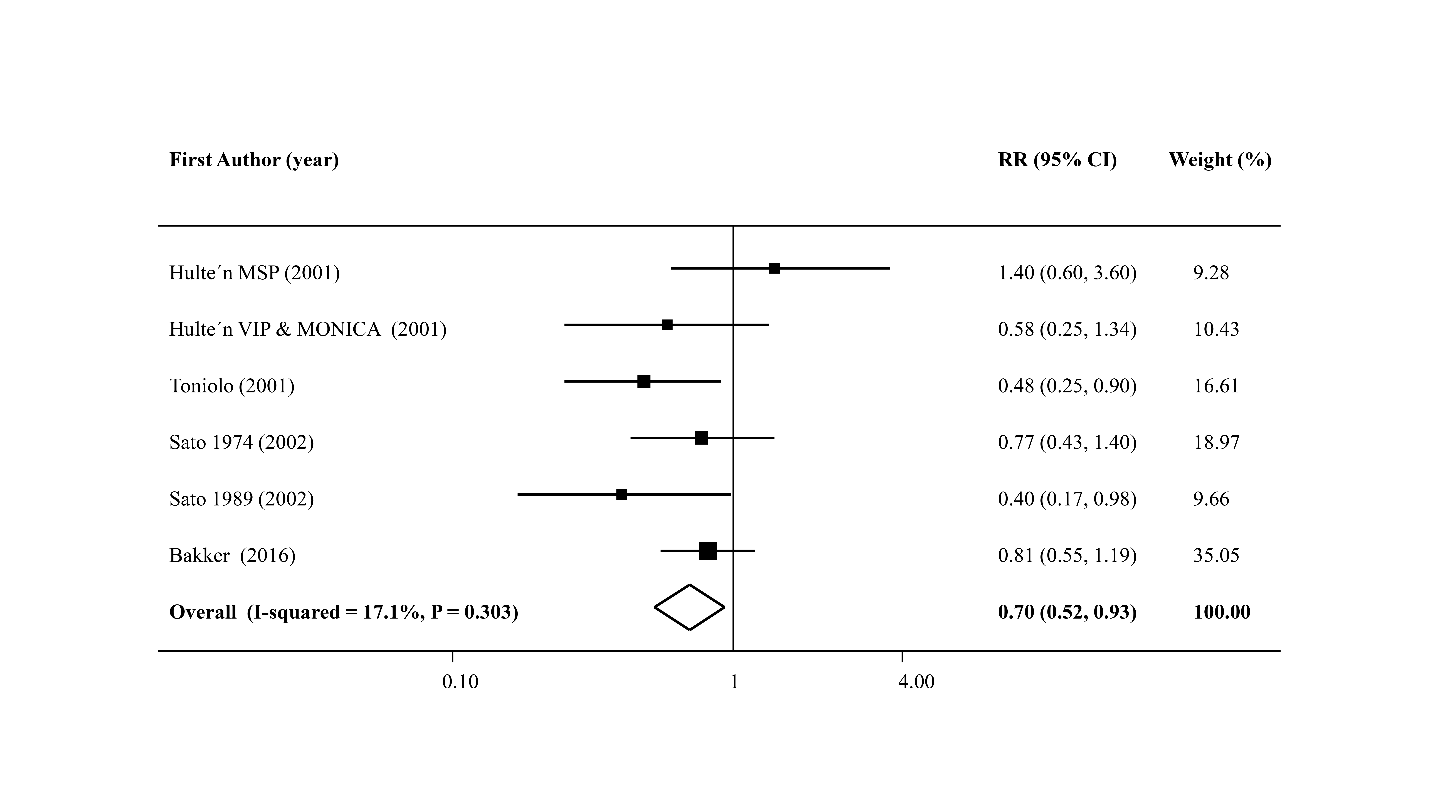
**Supplemental Figure 6A**. Forest plot for the association between circulating lutein and risk of breast cancer, expressed as comparison between highest and lowest categories of circulaing lutein. Diamonds represent pooled estimates from random effects analysis.


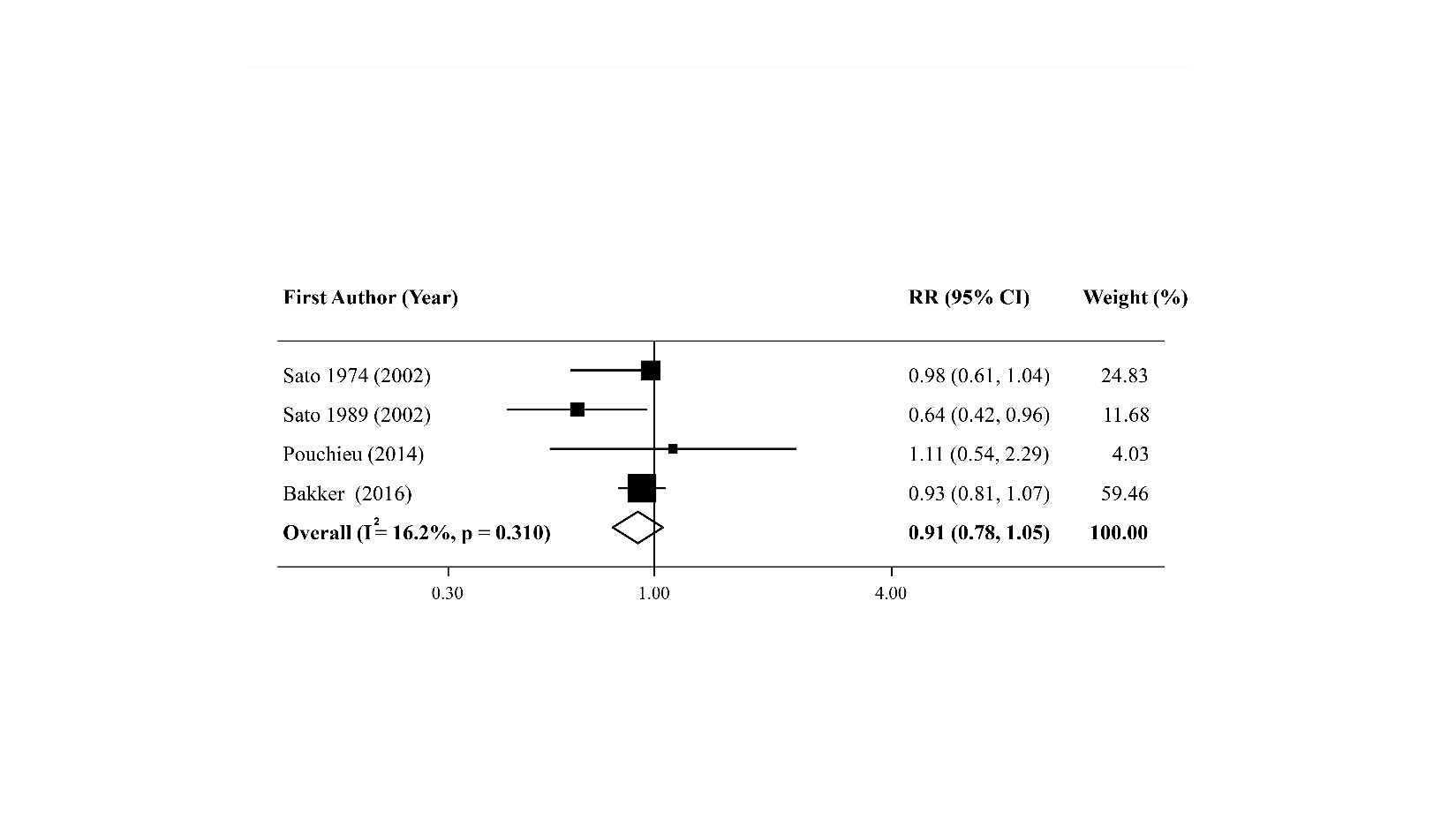
**Supplemental Figure 6B**. Linear dose-response analysis for the association between each additional 10 µg/dl of circulating lycopene and risk of breast cancer.


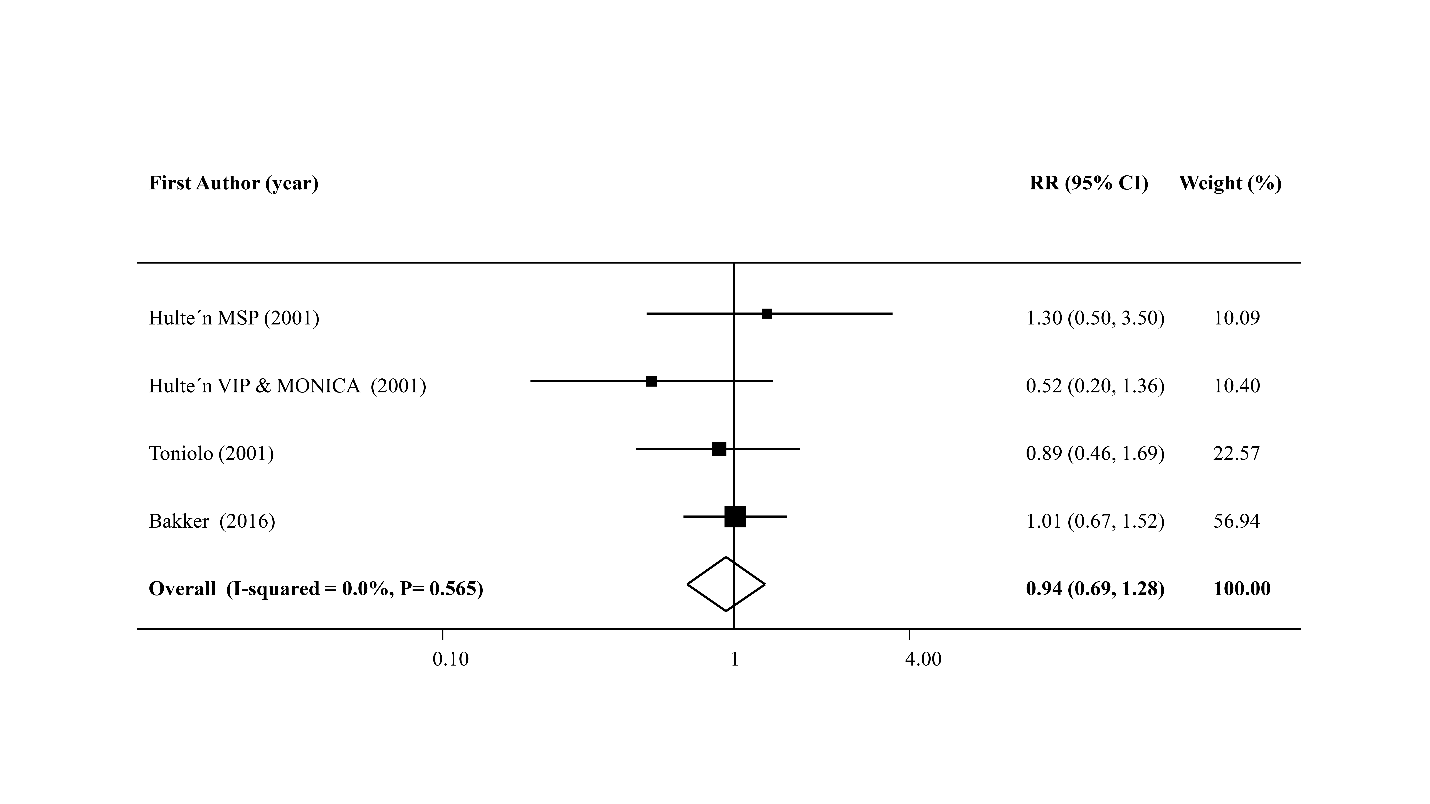
**Supplemental Figure 7A**. Forest plot for the association between circulating zeaxanthin and risk of breast cancer, expressed as comparison between highest and lowest categories of circulaing zeaxanthin. Diamonds represent pooled estimates from random effects analysis.


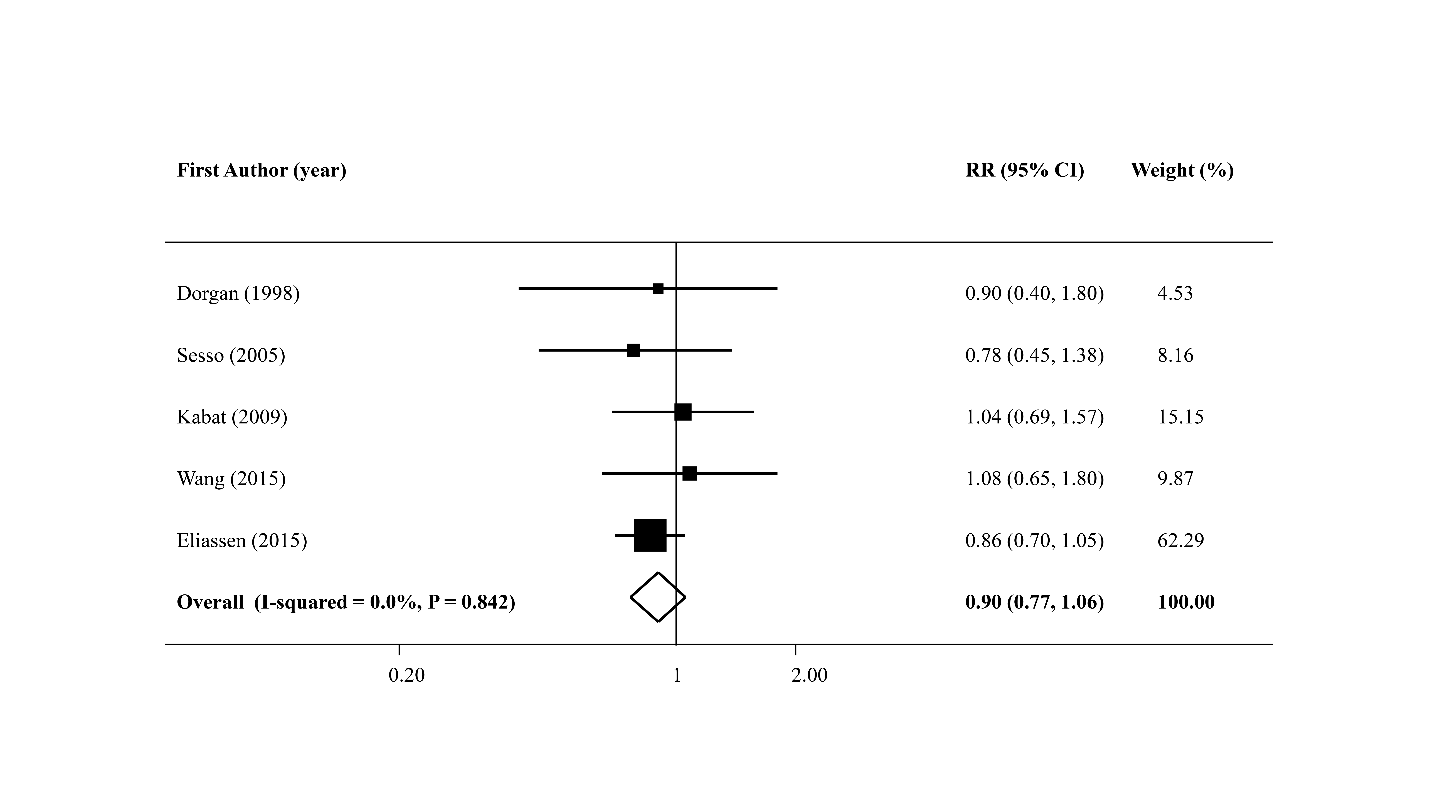
**Supplemental Figure 8A**. Forest plot for the association between circulating lutein/zeaxanthin and risk of breast cancer, expressed as comparison between highest and lowest categories of circulaing lutein/zeaxanthin. Diamonds represent pooled estimates from random effects analysis.


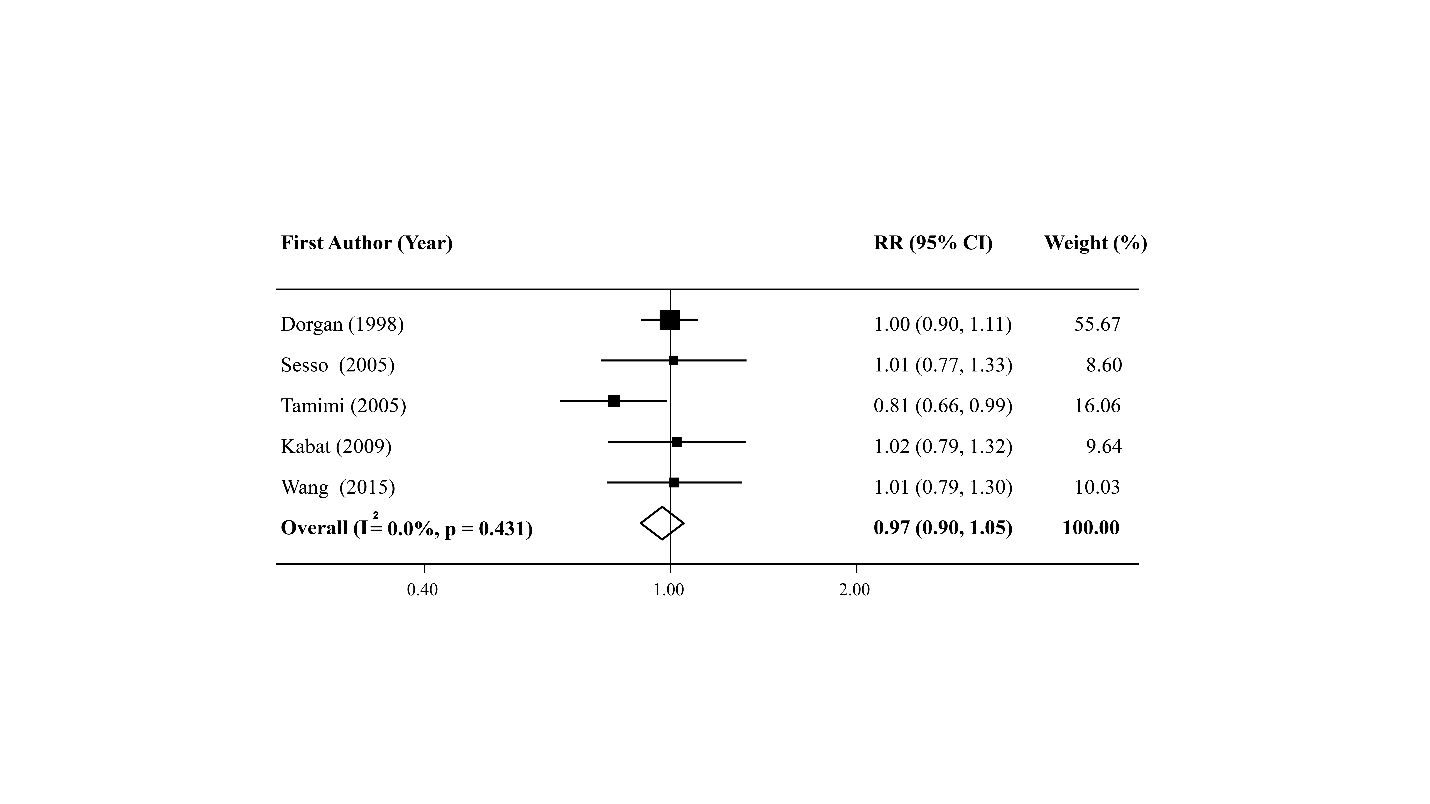


**Supplemental Figure 8B**. Linear dose-response analysis for the association between each additional 10 µg/dl of circulating lutein/zeaxanthin and risk of breast cancer.
